# Interplay of the Mass Transport and Reaction Kinetics for Lateral Flow Immunoassay Integrated on Lab-on-Disc

**DOI:** 10.3390/s25206271

**Published:** 2025-10-10

**Authors:** Snehan Peshin, Anthony Gavin, Nakajima Rie, Aarti Jain, Philip Felgner, Marc J. Madou, Lawrence Kulinsky

**Affiliations:** 1 Department of Mechanical and Aerospace Engineering, University of California, Irvine, CA 92697, USA; speshin@uci.edu (S.P.);; 2 Department of Chemistry, Saddleback College, Mission Viejo, CA 92692, USA; 3 Department of Physiology and Biophysics, School of Medicine, University of California, Irvine, CA 92697, USA; 4 School of Engineering and Science, Tecnológico de Monterrey, Monterrey 64849, Mexico

**Keywords:** centrifugal microfluidics, lateral flow assay, transport-reaction constant (TRc), surface reaction kinetics

## Abstract

**Highlights:**

**What are the main findings?**

- Introduced a new Transport Reaction Constant (TRc) that extends the traditional Damköhler number by incorporating film thickness, enabling better prediction of surface-based assay performance.
- Demonstrated that integrating lateral flow assays (LFAs) with a centrifugal Lab-on-Disc (LFA-CD) improves sensitivity by both increasing analyte dwell time at the test line and allowing for the increase of sample volume.

**What is the implication of the main finding?**

- TRc provides a generalizable design parameter for optimizing LFA and other surface-reaction assays under flow, complementing existing dimensionless numbers like Da.
- The use of LFA-CD platforms facilitates the increase in test sensitivity and lowering the limit of detection.
- The presented approach provides a scalable, low-cost pathway to prototype next-generation diagnostic devices whose microfluidic geometry and fluidic controls can be optimized based on the analytes’ reaction kinetics.

**Abstract:**

Lateral Flow Assays (LFAs) are ubiquitous test platforms due to their affordability and simplicity but are often limited by low sensitivity and lack of flow control. The present work demonstrates the combination of LFAs with centrifugal microfluidic platforms that allows for enhancement of LFAs’ sensitivity via the increase in the dwell time of the analyte at the test line as well as by passing a larger sample volume through the LFA strip. The rate of advancement of the liquid front in the radially positioned NC strip is retarded by the centrifugal force generated on spinning disc; therefore, the dwell time of the liquid front above the test line of LFA is increased. Additionally, integrating a waste reservoir enables passive replenishment of additional sample volume increases total probed volume by approximately 20% (from 50 μL to 60 μL). Comprehensive analysis, including COMSOL multiphysics simulation, was performed to deduce the importance of parameters such as channel height (100–300 μm), disc spin rate (0–2000 rpm), and reaction kinetics (fast vs. slow binding kinetics). The analysis was validated by the experimental observation of the slower-reacting CD79b protein on the test strip. For slower-reacting targets like CD79b, fluorescence intensity increased by ~40% compared to the static LFA. A new merit number, TRc (Transport Reaction Constant), is introduced, which refines the traditional Damköhler number (Da) by including the thickness of the liquid layer (such as the height of the microchannel), which affects the final sensitivity of the assays and is designed to reflect the role channel height plays for surface-based assays (in contrast to the bulk assays).

## 1. Introduction

Lateral flow assays (LFAs) are widely used point-of-care diagnostic devices known for their simplicity, affordability, and rapid time-to-answer. They operate without the need for complex instrumentation or trained personnel, making them particularly suitable for decentralized testing, home use, and low-resource settings. Usually, LFAs employ capillary action to transport the sample through a porous membrane, commonly made out of nitrocellulose (NC), where detection is achieved via a colorimetric or fluorescent signal generated by labeled conjugates binding to specific capture agents at test and control lines.

In a typical lateral flow assay (LFA), the sample flows by capillary action through a nitrocellulose (NC) membrane where analytes bind to specific capture molecules at designated test lines [1,2,3,4,5,6,7,8,9,10,11,12]. However, conventional LFAs have well-known drawbacks: result variability, relatively low sensitivity, small usable sample volumes, and no control over the flow rate once the assay commences [8,13,14]. Numerous formats and enhancements have been developed to improve LFA performance. For example, LFAs can be configured in competitive or sandwich formats and employ diverse labels (colored nanoparticles, fluorescent tags, etc.) to facilitate detection [15,16,17,18,19,20,21,22,23,24,25,26,27,28,29]. Isothermal amplification methods have enabled nucleic-acid based LFAs for genetic detection [30,31]. Despite these advances, the fundamental flow limitations of LFAs persist. To overcome these limitations, integrating LFAs with active microfluidic flow control has been explored [32,33,34,35,36].

One promising approach to the design of microfluidic tests includes utilization of Lab-on-Disc (LoD) platforms. A typical LoD has a format of a disc with incorporated reservoirs for a sample and reagents. When LoD is spinning, the centrifugal force pushes liquid from the center of the disc to its periphery and liquid propulsion, sample metering, mixing, valving, and various other sample preprocessing and processing steps can be performed without the use of external pumps. LoD platforms have been widely applied for point-of-care diagnostics, owing to their compact format, low cost, and the ability to automate multiple assay steps on systems that can be isolated, limiting possibility of infection and cross-contamination [37,38].

**Figure 1:**
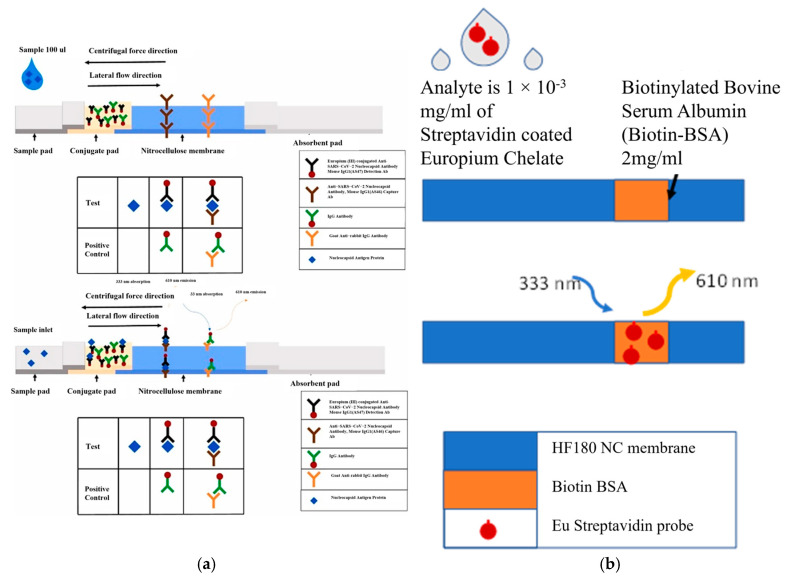
(**a**) Schematic of the lateral flow assay integrated on centrifugal LFA-CD device used in this work, showing the sample inlet, nitrocellulose membrane strip (NC) with test line, and the adjacent waste reservoir integrated on a spinning disc platform. (**b**) Schematic of the simulation model, featuring a test line of biotinylated bovine serum albumin (BSA–biotin) (approximately 2 mg/mL) printed on an NC membrane (~0.2 mm thick, 6.5 μm pore size, porosity 0.8; Millipore HF180). This model geometry was used in COMSOL 6.0 to simulate the capillary flow and reaction kinetics in the system (see text for details). Disc spin rates reported as rpm and corresponding front velocity u (m/s), derived from time-to-line measurement.

In this work, we incorporate an LFA strip with a centrifugal LoD platform—a rotating Compact Disc (CD)—to modulate the flow. Figure 1a shows a schematic of the integrated LFA-CD device used in this study, depicting the NC membrane strip mounted on a rotating disc along with its sample inlet and waste reservoir [32]. By spinning the disc, capillary flow in the membrane can be slowed or halted as needed, increasing the analyte’s dwell time at the test line and enabling multiple sample replenishment steps. This approach allows a greater volume of sample to interact with the test line over time, thereby improving the assay’s sensitivity and lowering its limit of detection. Centrifugal microfluidic integration of LFAs has been reported by several groups [33,34,39]. For instance, Wiederoder et al. achieved an order-of-magnitude lower detection limit than a standard LFA by combining paper microfluidics with a spinning disc to pre-concentrate the sample and control flow via rotation. Kainz et al. [34,39] demonstrated that directing flow radially through the membrane under centrifugal force allows independent control of flow rate and incubation time, improving the quantitative consistency of the assay. Shen et al. [35,36] introduced a bent-strip LFA-on-CD design (eliminating the need for a traditional absorbent pad) to accommodate larger sample volumes, which significantly boosted signal intensity [40]. In the present work, we build on these innovations by combining active flow control via disc spinning (as in our prior work) with the bent-strip design from Shen et al. [35,36]. This combined approach maximizes both the available reaction time and the total sample volume processed in a single assay.

Meanwhile, theoretical models have been developed to better understand and optimize LFA performance. Ragavendar et al. [41] used simulations to optimize test line placement, sample volume, and reaction time for improved sensitivity, and Qin et al. formulated a model to explain and mitigate the “hook effect” (signal reduction at high analyte concentrations) in LFAs [41]. However, these studies considered traditional LFAs without externally controlled flow. Here, we extend the analysis to flow-controlled LFAs by applying the Merril–Millipore equation [42] to relate membrane capillary flow and applied centrifugal forces.

We recognize that existing metrics like the Damköhler number Da (the ratio of reaction rate to advective transport rate) used in prior LFA studies [43] do not account for the finite thickness of the flow channel [44,45]. Therefore, we introduce a new dimensionless parameter, the Transport Reaction constant (TRc), which incorporates the microchannel height (fluid film thickness) into the reaction–transport analysis. Unlike Da (originally formulated for well-mixed bulk reactions), TRc captures the interplay between reaction kinetics and mass transport in surface-bound assays with limited thickness (such as LFAs). In the limit of very shallow channels (channel height h on the order of the reaction-diffusion penetration depth δ_m_), TRc ≈ Da, and conventional kinetics analysis is adequate. In deeper channels or higher-flow conditions, where only a fraction of the analyte interacts at the surface before being carried away, TRc is significantly lower than Da, indicating a greater influence of transport limitations. We identify three mass transport–reaction rate regimes: TRc > 1 (reaction-rate–limited, fast transport), TRc < 0.1 (transport-limited), and 0.1 < TRc < 1 (a transitional regime where both transport and reaction affect performance). This framework provides an insight into LFA behavior across different geometries and flow conditions, guiding the optimization of assay design in LFA-CD systems and other microfluidic assay platforms.

Although centrifugal flow control for LFAs has demonstrated timing enhancement and sensitivity improvements [34,36,46], prior work did not recast reaction–transport performance in a metric that indicates the deficiencies of thick liquid films and short residence times. The new merit number, TRc, closes this gap and yields actionable regime maps guiding geometry and operating conditions of such microfluidic surface-based assays. The detailed formulation of TRc is described in Section 4 below.

## 2. Materials and Methods

### 2.1. Fabrication of the LFA-CD

The lateral flow assay-integrated Lab-on-Disc (LFA-CD) was fabricated at the BiNoM Laboratory, University of California, Irvine, (Irvine, CA, USA) using precision micromachining and membrane integration techniques. The finalized LFA-CD platform comprises four primary layers: an acrylic base disc (CNC-milled), a Nitrocellulose (NC) membrane strip, a membrane-bonding adhesive layer, and top and bottom sealing adhesive layers.

The base disc was designed using SolidWorks 2023 (Dassault Systèmes, Vélizy-Villacoublay, France), and the design files were used to machine poly(methyl methacrylate) (PMMA) sheets (McMaster-Carr, Elmhurst, IL, USA; product #8589K43, Clear Scratch- and UV-Resistant Acrylic Sheet) using a Tormach 440 PCNC CNC milling machine (Tormach Inc., Waunakee, WI, USA). The front side of the disc features a machined membrane chamber and a waste reservoir, while the backside accommodates a circular sample inlet well and provides structural cutouts for flexibility. A narrow through-hole is milled to accommodate the bending of the membrane strip, enabling fluidic flow across its length.

The membrane chamber slot was milled to be 3.2 mm wide to snugly fit standard 3 mm-wide NC strips, and the slot depth was varied (100 μm, 200 μm, or 300 μm) to study the influence of liquid film thickness on assay performance.

The NC membrane (Hi-Flow Plus HF120, Millipore Sigma, Burlington, MA, USA) was cut to ~40 mm length using a Silhouette Cameo 4 Electronic Cutter (Silhouette America, Lindon, UT, USA). A Biodot AD3050 system (BioDot Inc., Irvine, CA, USA) was used to dispense antigen capture lines directly onto the NC membrane before integration into the cartridge.

To affix the membrane into the chamber, we used a double-sided pressure-sensitive adhesive compatible with lateral flow membranes (AR #90445Q; Adhesives Research, distributed by Parafix Tapes & Conversions Ltd., Lancing, UK). The entire fluidic chamber (both front and back) was then sealed with single-sided 3M pressure-sensitive sealing tape (9795R, Saint Paul, MN, USA) to protect the fluid path and maintain channel confinement.

The analyte inlet was realized by machining a dedicated circular inlet reservoir (depth ~1 mm) on the back of the CD directly above the NC membrane chamber. This inlet connects vertically through a via hole to allow the deposited sample to contact the beginning of the NC strip, with venting channels designed to prevent bubble formation and enable consistent flow.

Finally, to ensure complete sealing and uniform adhesion, a roller (Akiro B07YDNKSH6, Amazon, Seattle, WA, USA) was used to apply even pressure over the disc during final assembly. Both the inlet and membrane chambers were vented to atmospheric pressure to permit uninterrupted fluid flow.

**Figure 2:**
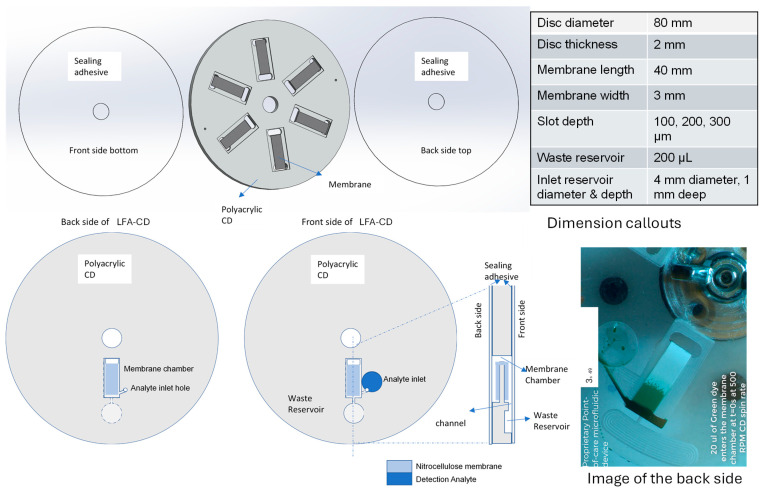
Illustration of the assembly of the lateral flow LFA-CD. The top set of sketches demonstrates the three layers of the assembled CD: the backside adhesive, the CNC-machined polyacrylic layer, and the frontside adhesive layer. The table of critical geometric dimensions of the system is provided. The bottom row of sketches illustrates components of the disc as viewed from below the disc (back side of CD), from above (front side of CD) as well as the disc’s cross-section. The photo of the top side of the disc is provided on the bottom right side inset.

Figure 2 illustrates the assembly of the CNC-milled disc with the embedded NC strip. The LFA-CD disc, fabricated from PMMA sheet, has a diameter of 120 mm and a thickness of 2 mm. The nitrocellulose strip integrated into the disc is 40 mm long and 3 mm wide, housed in 3.2 mm wide milled slot. The slot depth was varied from 100 µm to 300 µm to conduct experiments for different liquid film heights. The analyte inlet reservoir has a diameter of ~4 mm and a depth of 1 mm, holding a sample volume of ~25–50 µL, while the waste reservoir holds liquid volume of 200 µL.

The LFA-CDs were fabricated using the following steps:

1. CAD Design and CNC Milling: The disc was designed in SolidWorks and machined from 3 mm-thick polyacrylic (PMMA) sheets using a Tormach 440 PCNC Mill (Tormach Inc., Waunakee, WI, USA). The features included
  - A 3.2 mm-wide membrane chamber (depth varied: 100–300 µm);
  - Circular inlet well (~1 mm deep);
  - Waste reservoir;
  - Through-hole for membrane bending.
2. Membrane Preparation: Hi-Flow Plus HF120 (Millipore Sigma, Burlington, MA, USA) (3 mm × 40 mm) were cut using a Silhouette Cameo 4 (Silhouette America, Lindon, UT, USA); detection lines dispensed using BioDot AD3050 (BioDot Inc., Irvine, CA, USA).
3. Membrane Integration: NC membranes were affixed to the chamber using double-sided lateral-flow adhesive from Adhesives Research #90445Q (distributed by Parafix Tapes & Conversions Ltd., Lancing, UK).
4. Sealing: Single-sided 3M 9795R single-sided tape (3M Company, Saint Paul, MN, USA). was used to seal the top and bottom surfaces of the fluidic disc.
5. Inlet Channel Connection: A via hole was machined to connect the inlet well to the start of the NC strip, allowing sample introduction.
6. Final Assembly and Venting: A pressure roller Akiro Manual Roller B07YDNKSH6 (Amazon USA) was used to ensure uniform sealing. Vent holes were added to inlet and outlet chambers for pressure equalization.

### 2.2. Experimental Setup

**Figure 3:**
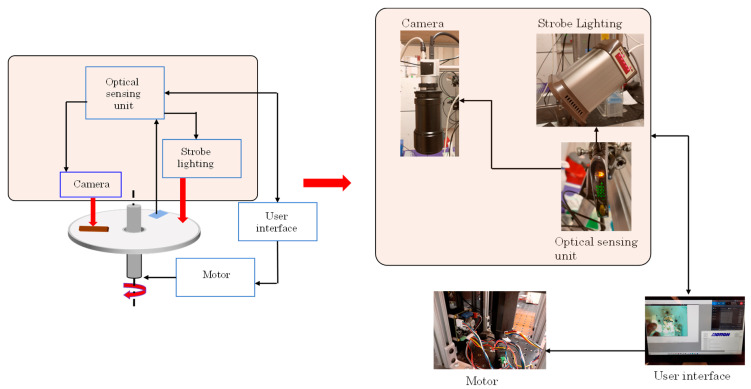
Experimental setup for the centrifugal LFA platform. Key components of the system include the spin motor and chuck holding the LFA-CD device (disc), a stroboscopic LED coupled with an optical sensor used to monitor the disc’s rotation speed (via a reflective marker on the disc edge), and a high-speed camera positioned above the disc.

A custom spinstand with integrated controllers, stroboscopic light source and a camera was assembled as illustrated in Figure 3. The CD is secured onto a custom chuck that is rotated using a brushless DC (BLDC) servo motor (SM3450D, Motion USA, Columbus, OH, USA), which is controlled via a BLDC servo motor controller (EZSV23/EZSV17, AllMotion, Union City, CA, USA). A custom Python 3.10 interface (Python Software Foundation, Wilmington, DE, USA) was developed using serial communication (RS-232/USB) to send commands to the motor controller via AllMotion’s command set containing angular velocity, acceleration, and timing parameters. To monitor the actual spin speed of the CD during operation, a small reflective piece of aluminum foil is affixed to the edge of the disc and tracked using an optical sensor. The light issued from the LED located above the disc is backscattered to the sensor and the time between two sequential light pulses allows for calculation of the actual angular velocity of the disc [47]. The light backscattered from the foil also triggers the strobe lighting (DT-311A, Shimpo, Lynbrook, NY, USA) and a shutter of the digital camera (acA800-510uc, Basler AG, Ahrensburg, Germany). The series of images are saved as a video using a screen recording software Bandicam (Bandicam Company, Irvine, CA, USA).

The system logged the number of revolutions per minute (RPM) with a resolution of ±10 RPM and temporal sampling of 1 Hz. Repeated measurements (n = 5 per RPM setting) showed a coefficient of variation (CV) < 2.5%. These measurements were compared to the BLDC servo motor controller readout (EZSV23/EZSV17, AllMotion, Union City, CA, USA), demonstrating agreement (±2% error) across RPM settings ranging from 0 to 3000 RPM.

### 2.3. Lateral Flow CD Design and Operation

The schematics of a lateral flow immunoassay are depicted in Figure 1. The testing utilizes Fluoro-Max fluorescent streptavidin-coated particles (29470701010350, Thermofisher Inc., Waltham, MA, USA) as the analyte probe, with analyte concentrations (diluted in Phosphate-Buffered Saline (PBS) (10× pH 7.4, RNase-free, AM9624, Thermofisher Scientific, Waltham, MA, USA) ranging from 1 × 10^−3^ to 1 × 10^−4^ mg/mL. The design of the CD with the integrated lateral strip is presented in Figure 2. The sample is loaded through the analyte inlet hole, the inlet is sealed, and the disc is flipped (so that the analyte inlet is now on the bottom of the disc) and placed on the spin chuck, with the waste reservoir positioned adjacent to the top of the disc. Having the waste reservoir on the top of the disc facilitates the detection of the completion of the testing by evaluating how full the waste reservoir is. The analyte inlet is located at the backside of the disc, with a drilled hole connecting it to the top of the disc. In addition to the main design of the disc utilized in the present study (Figure 2), two alternative disc designs were created and are detailed in the Supplementary Materials (Section S1).

The sequence of process steps include

1. Pipetting 20 μL of analyte (Streptavidin coated Polystyrene particles with Europium) into the analyte inlet reservoir.
2. Spinning the disc at 500, 1000, 1500 or signal enhancement specified rpm (see Section 5.2) for 2 min.
3. Stopping the disc.
4. Spinning the disc at 5000 rpm in order to squeeze out the fluid from the section of NC membrane bent to the back side of the CD. Draining the section of NC strip restores the ability of the NC membrane to pull up more liquid samples.
5. Adding more volume of analyte via analyte inlet reservoir and proceeding from step 2 on. It is possible to go through steps 2–5 multiple times until the NC membrane is soaked through and no further capillary action is possible. Three of these cycles were performed and additional two sample volumes of 20 μL each (for the total 60 μL of sample) were processed.

After the process is complete, the strip is read in Tiny Imager with a UV light LED with an emission spectrum of 365 nm and a sensor accepting the light with the wavelength range of 600–630 nm.

Front velocity u (m/s) was measured directly using a synchronized strobe imaging setup with 3–5 replicates for each selected angular disc velocity. Spin speeds of 500, 1000, and 1500 RPM were selected to represent low, moderate, and high centrifugal forces, corresponding to centrifugal forces sufficient to slow down the capillary flow rather than stopping it completely.

### 2.4. Fluorescent Probes and Detection Method

**Figure 4:**
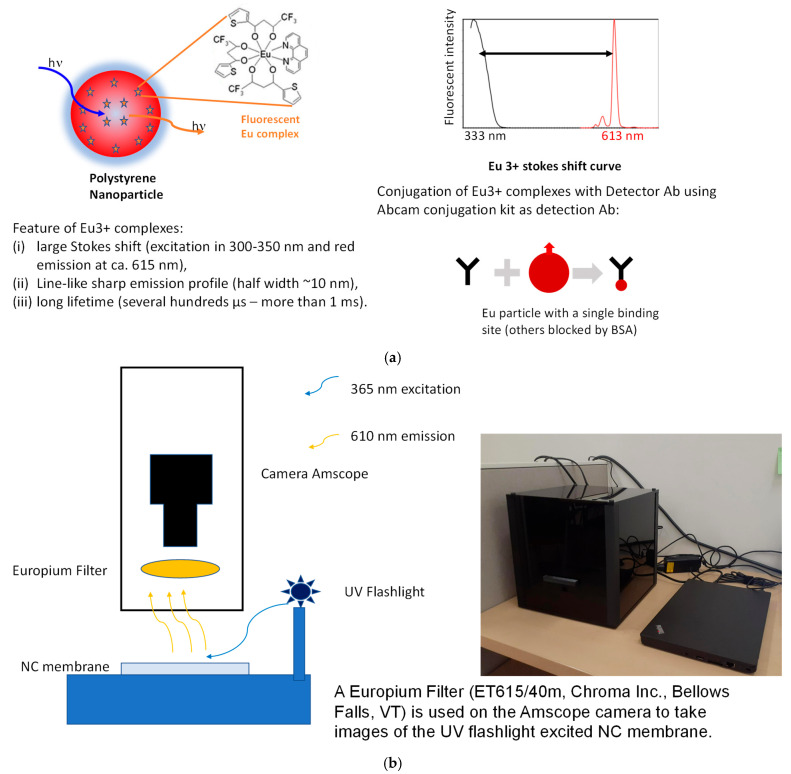
(**a**) Europium chelate-based fluorescent tag used as the detection probe in the assay (Fluoro-Max streptavidin-coated Eu microparticles binding to biotinylated targets). (**b**) Schematic (left) and photograph (right) of the custom-built “Tiny Imager” device used to detect the fluorescent signal from the NC membrane’s test line. The Tiny Imager consists of a compact fluorescence camera and optics, capturing the emitted light from the test line. Arrows represent light with specific wavelengths.

The fluorescent probes consist of polystyrene nanospheres with Eu fluorescent complex on their surface as shown in Figure 4a. These nanospheres are bio conjugated to the antibodies. For biotin–streptavidin interaction, Fluoro-Max fluorescent Streptavidin-coated particles and BSA biotin printed strips (Catalog number: 29470701010350, Thermo Fisher Scientific, Waltham, MA, USA) are used. This is a well-established model system with fast and high-affinity binding kinetics, making it ideal to validate platform sensitivity and fluidic response. For Eotaxin the recombinant Anti-Eotaxin antibodies (ab133604) and recombinant Human Eotaxin proteins (ab282376) (Abcam, Cambridge, UK) are employed. Eotaxin represents a moderate-affinity cytokine interaction relevant in inflammation and allergy diagnostics. For CD79b the recombinant Anti-CD79b antibody (ab134103) and Recombinant Human CD79b protein (ab153795) (Abcam, Cambridge, UK) are utilized and were selected to represent slower, weaker-binding protein–antibody interactions, commonly observed in cancer or immune biomarker detection. The bioconjugated antibodies for Eotaxin and CD79b are conjugated to Europium by using Europium conjugation kit [48] (utilizing the recipe provided by the kit manufacturer—see Supplementary Section S2). These three systems collectively span the range of fast to slow binding kinetics, allowing evaluation of how flow rate and membrane thickness affect both signal development and Transport Reaction Constant (TRc) sensitivity across different reaction regimes. Streptavidin coated Europium dilutions in Phosphate-Buffered Saline (PBS(10× pH 7.4, RNase-free, Catalog number: AM9624, Thermo Fisher Scientific, Waltham, MA, USA) advance over the NC strips coated with biotin BSA. The Europium complex is excited by 300–350 nm light and it emits at 615 nm wavelength. The advantage of this probe is that it has a large Stokes shift [49]. There is a line-like sharp emission profile (half width~10 nm). It also has a long lifetime from several hundred μs to more than 1 ms. This probe is conjugated with antibody of choice or comes coated with Streptavidin. The conjugation of Eu complex to a detector Ab is accomplished using Abcam conjugation kit.

Several species (biotin–streptavidin, Eotaxin, and CD79b) with different dissociation constants (Kd) were tested:

- Biotin–streptavidin: Kd = 10^−15^ M (Fastest Reacting)
- Eotaxin: Kd = 7.1 × 10^−11^ M (Moderate Reaction)
- CD79b: Kd = 3.9 × 10^−9^ M (Slowest Reacting)

In order to measure the signal at the test line, after the disc is spun, the top adhesive layer is detached and the NC membrane is removed from the disc. The NC membrane is then placed into the custom-made Tiny Imager (see Figure 4b) that consists of the Amscope camera [Amscope MU2003/ET615/40m] with the attached Europium filter and the UV flashlight (365 nm) [UV Flashlight, Temu Inc., Boston, MA, USA] enclosed within a box and run with custom software. The camera takes pictures of the test line of the NC membrane and this image is processed using ImageJ version 1.53 software to calculate the gray scale of the signal.

For each experimental condition, three independent replicates (n = 3) were tested. The fluorescence intensity at the test line was quantified using the Tiny Imager’s built-in analysis software, and error bars representing the standard deviation across replicates are shown in relevant plots. The observed variation across replicates was <10% relative standard deviation.

#### Antibody–Europium Conjugation and Quality Control

Antibodies (Eotaxin, CD79b) were Eu-labeled per kit protocol (see Section S2). Post-conjugation, the unconjugated dye was removed by size-exclusion, while protein was quantified by BCA (Bicinchoninic Acid assay) and Eu was quantified by absorbance. The hydrodynamic size and ζ-potential shift were confirmed by DLS (dynamic light scattering) to verify successful coupling. Conjugation efficiency (% protein labeled) and final working concentration (mg/mL; n = 3 preps) are reported in Section S3. Across all antibody-Eu conjugates, conjugation efficiency averaged 72.3 ± 5.1%. Calibration curves for the analytes’ detection are provided in Section S4. The detailed list of information for models and manufacturers of various materials, supplies, and equipment utilized in this study is provided in Section S5.

## 3. COMSOL Simulation

The capillary flow in the nitrocellulose membrane on a rotating CD was modelled using COMSOL simulation software version 6.0 [50]. The Darcy’s law module, phase transport in porous media module with water and air as multiphase laminar flow, and multiphase flow in a porous medium module were employed in addition to the main program framework. For modeling the reaction kinetics, the reaction engineering module was utilized and data for the concentration of species vs. time was collected. A nitrocellulose membrane was represented in a two-dimensional simulation, by a strip with a width of 2 mm and a length of 4 cm. The test line (with surface immobilized species) that is 1 mm wide, is located on top of the NC and it is 2 cm away from the edge of the NC membrane that is near the location where the analyte is introduced. The outlet is located at the distal end of the NC membrane. The effect of the rotation of the CD spin rate was modelled by varying the outlet pressure condition of Darcy’s law.

The model employs a sandwich baseline acquired from COMSOL [50] (Comsol Multiphysics, Burlington, MA, USA). Figure 1b presents the schematics of the model featuring a test line comprising BSA-biotin (Bovine Serμ Albumin) at approximately 2 mg/mL, printed on an NC membrane with a thickness of 0.2 μm, a pore size rating of 6.5 μm, and porosity of 0.8, designated as HF 180 (Millipore Sigma, Burlington, MA, USA).

For a lateral flow on rotating disc, the centrifugal force (in addition to the capillary force reflected in Darcy’s Law) exerts a major influence on the mass transport of the liquid sample in the membrane.

The pressure exerted on the liquid sample by the centrifugal forces is represented by [47]

(1) $$P_{r}=\frac{\rho \omega^{2}∆r\vec{r}}{A},$$

where $P_{r}$ is the pressure inside the liquid sample located the distance r from the center of the disc; *ω* is the angular velocity of the disc; the average radial distance of the center of the flowing fluid in the channel is $\vec{r}$; and the height of the fluid column in the radial channel is ∆r; and the density of the fluid flowing in the channels is *ρ*.

Darcy’s law is a simple proportionality relation of instantaneous flux $q=\frac{Q}{A}$ [units of m/s] with permeability k of the porous medium, the dynamic viscosity µ, the pressure drop Δp over a given lateral flow strip length given by the following relations [51]:

(2) $$q=\frac{-k}{µL}\Delta p,$$

(3) $$k=\frac{por}{8{Rc}^{2}},$$

where por is the porosity of the lateral flow strip and Rc is the pore size of the lateral flow strip.

**Figure 5:**
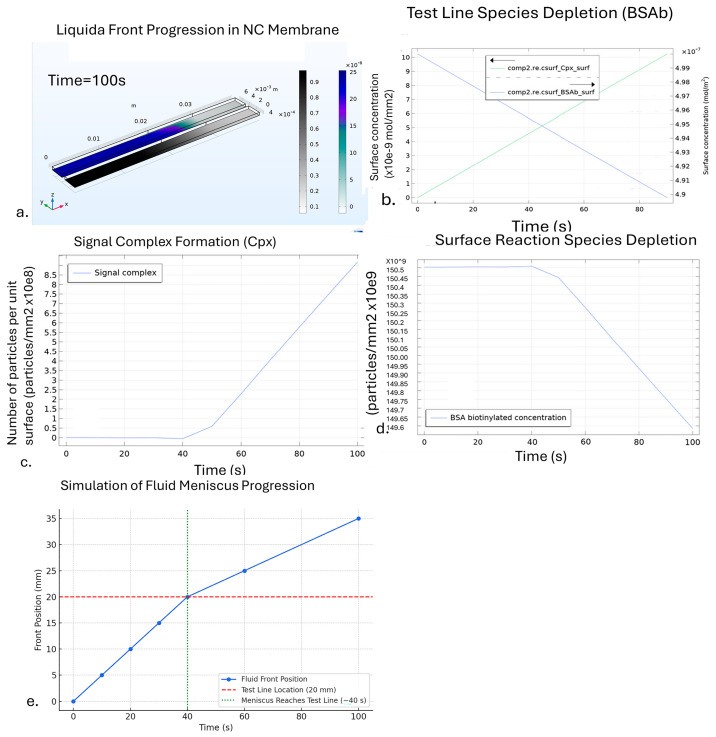
COMSOL simulation results illustrating flow and reaction dynamics in the LFA−CD system. (**a**) Cross-sectional view of fluid front propagation through the NC membrane. The gray region shows water flow, and the blue region represents Europium-labeled analyte distribution at 100 s. (**b**) Temporal evolution of surface concentrations at the test line: the biotin–BSA binding site concentration decreases (green), while the concentration of signal complex (blue) increases, indicating ongoing binding. The arrows indicate the opposing reaction trends—**the green arrow** marks the decline in the surface concentration of immobilized BSA–biotin (BSAb) as it is consumed during the binding reaction, while the blue arrow highlights the corresponding increase in signal complex (Cpx) formation at the test line. These trajectories intersect around 80 s, representing the progression toward reaction equilibrium. (**c**) Growth of signal complex over time, represented as the number of particles per unit surface. (**d**) Depletion of biotinylated BSA sites at the test line, showing near−constant concentration until ~40 s, followed by steady decline. These panels collectively illustrate how analyte transport and reaction kinetics evolve in the simulation. Simulations performed with flow velocities corresponding to rpm values; conversion to u (m/s). (**e**) Simulation of fluid meniscus progression along the nitrocellulose (NC) membrane.

The results of the COMSOL simulation, depicted in Figure 5, provide a comprehensive view of the mass transport and reaction dynamics within the system over a 100-s period. Figure 5a demonstrates the fluid’s movement through the nitrocellulose membrane, where the advancement of the liquid front is illustrated by the extension of the gray region, representing water, contrasted with blue, indicating Europium reactants in fluid. In Figure 5b, the reaction progression is reflected, showing the decline in test line species concentration and the concurrent increase in signal complex formation over time. Figure 5c visualizes the formation of the signal complex as the analyte is transported to the test line, leading to the reaction products forming the signal complex. The underlying reaction is represented as

(4) EuStr+BSAb(ads)=>Cpx(ads),

where EuStr is the analyte Europium Streptavidin which is detected at the test line; the BSAb(ads) is the biotinylated Bovine serum albumin species immobilized at the test line; and Cpx(ads) is the product called the signal complex. Figure 5d demonstrates gradual reduction in the concentration of biotinylated BSA as it reacts with Europium Streptavidin. The concentration remains unchanged during the initial period up to 40 s because it takes that long for the fluid meniscus to reach the test line.

At equilibrium, Equation (4) is written as

(5) $$EuStr+BSAb(ads)\overset{Ka/Kd}{↔}Cpx(ads).$$

The dynamic equilibrium equation can be stated as

(6) $$K_{on}\left[EuStr\right]\left[BSAb\right]\left(ads\right)=K_{off}\left[Cpx\right](ads).$$

where $K_{on}$ is the equilibrium reaction constant for the forward direction and $K_{off}$ is the equilibrium reaction constant for the reverse direction. Brackets indicate the concentration of the various reactants and products with (ads) indicating means surface concentration of the indicated species.

(7) $$K_{a}=\frac{K_{on}}{K_{off}}=\frac{\left[Cpx\right]\left(ads\right)}{\left[EuStr\right]\left[BSAb\right]\left(ads\right)}and,\;K_{d}=\frac{K_{off}}{K_{on}}=\frac{\left[EuStr\right]\left[BSAb\right]\left(ads\right)}{\left[Cpx\right](ads)},$$

where $K_{a}$ is the association constant and $K_{d}$ is the dissociation constant.

Figure 5e presents the simulation results of the advancement of the fluid along the NC membrane from the sample deposition end of the test strip. The test line is 20 mm distant from the inlet where the sample is deposited. The simulated fluid front reaches this test line at approximately 40 s, beyond which surface-bound analyte interactions initiate. This result is consistent with the delay observed in species depletion and signal complex formation (Figure 5b–d).

To evaluate the parametric dependence of the system on various variables, a set of simulations used specific parameters corresponding to the experimental setup. The parameters considered included the volume of sample fluid, pore size of the NC membrane, and adjustments to *K_on_* and *K_off_* modifying reaction kinetics and adsorption coefficients. This approach was aimed at determining whether adsorption, rather than mass transport, is the limiting factor. The results indicated no significant difference between heterogeneous and homogeneous scenarios. Additionally, a rotating frame was introduced to modify the flow rate. Table 1 below lists parameters utilized in the discussed model.

**Figure 6:**
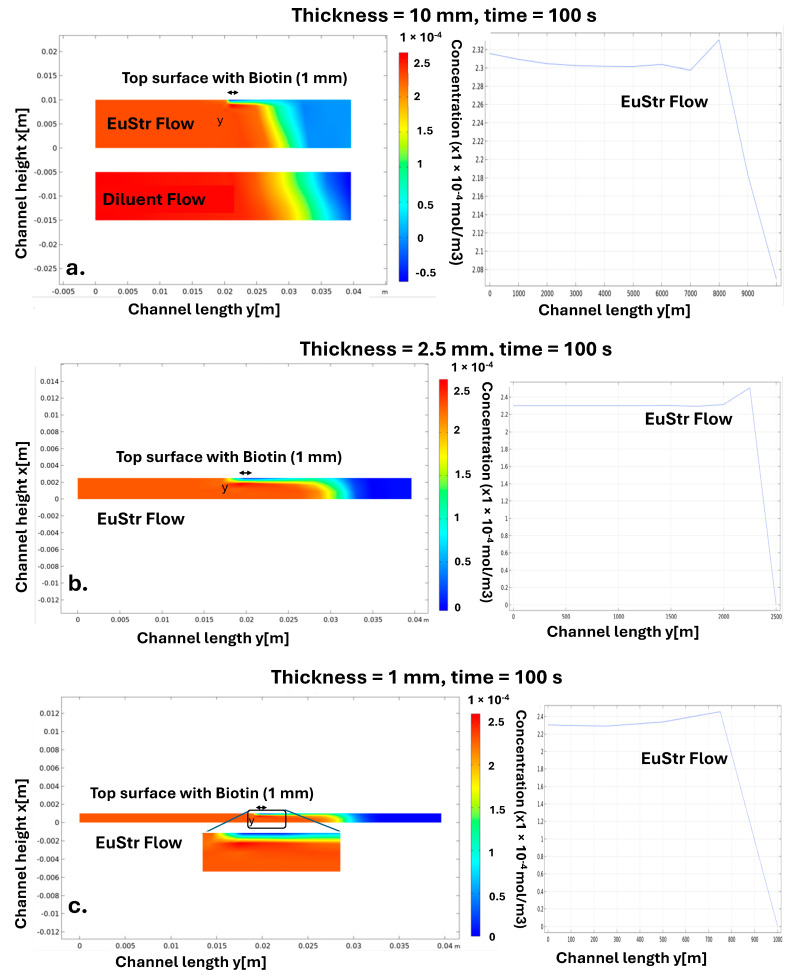

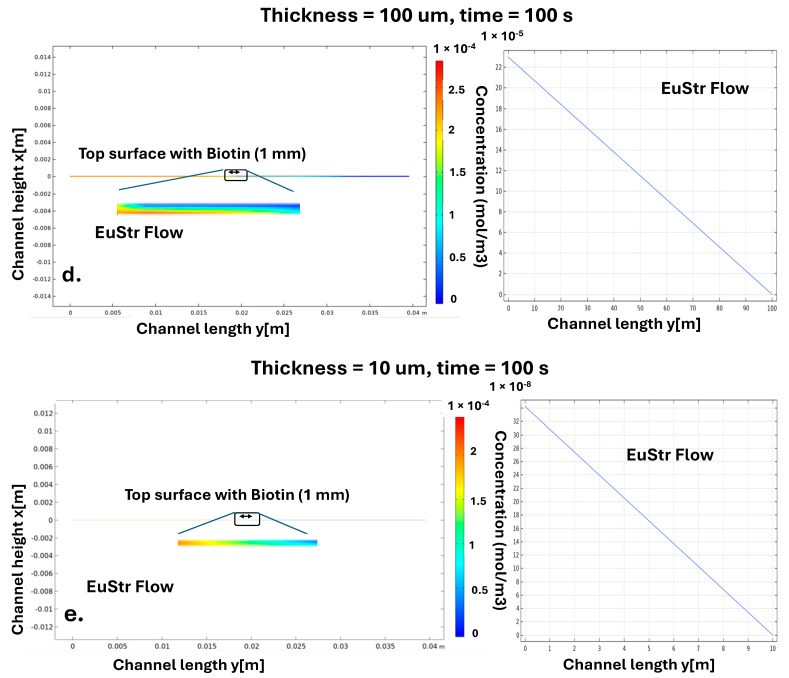
Simulation results of unreacted Europium-conjugated particles shown for the cross-section of the paper strips (membranes) of various thicknesses. The test lines (1 mm wide) on the top surface of the membranes are indicated by double-headed arrows. The vertical distance from the top of the membrane is provided on the vertical axis of the plots. The Europium-conjugated particles are reacting with the biotin molecules on the test line and the particle concentration above the test line becomes small (represented by the blue color of the plots). Because the simulation run represents 100 s, capillary force pulls the sample to about 30–40 mm along the length of the membrane and thus the blue color beyond 40 mm signifies regions of the membrane that are not reacted yet. Gradual change of color between red and blue indicates the diffusional transport of the particles creating concentration gradients. The plots illustrate the concentration profiles of (ii) Europium and (iii) the reaction product across different film heights: (**a**) 10 mm (the “diluent flow” result indicates the propagation of the water without beads through the membrane), (**b**) 2.5 mm, (**c**) 1 mm, (**d**) 100 microns, and (**e**) 10 microns. Arrows indicate the direction of Europium–streptavidin (EuStr) flow and the biotin-coated surface region (1 mm) where reaction occurs.

Figure 6 illustrates the simulation results for unreacted Europium-conjugated particles, presenting a cross-sectional view of the paper strip’s concentration profile for membranes of different thicknesses. For each membrane thickness, the concentration profile of the particles across membrane height is shown, starting with a membrane thickness of 10 mm in Figure 6a. The Europium-conjugated particles are reacting with the biotin molecules on the test line and the particle concentration above the test line becomes small (represented by the blue color of the plots). Because the simulation run represents 100 s, capillary force pulls the sample to about 30–40 mm along the length of the membrane and thus the blue color beyond 40 mm signifies regions of the membrane that are not reacted yet. Gradual change of color between red and blue indicates the diffusional transport of the particles creating concentration gradients. It is instructive to compare the concentration change of Europium beads containing flow with propagation of just water without beads (where concentration of the advancing solution is not reduced at the test line). It can be seen that as the membrane thickness to 2.5 mm (see Figure 6b), to 1 mm (Figure 6c) and to 100 microns (Figure 6d), the changes in the concentration of the particles near the test line are becoming comparable with the height of the membrane. When the membrane is 10 micron thick (Figure 6e), the depletion of the particles (resulting from the reaction taking place at the test line) propagates through the whole thickness of the membrane. Therefore, we can see that while there will be a little difference between the membranes that are 10 mm thick or 2.5 mm thick (in these cases, the concentration profile above the test line would emulate infinitely thick membrane), when the membrane is much thinner (below 100 micron thick), the boundary layer above the test line where the concentration of the analyte is changing is comparable with the membrane thickness.

These results suggest that channel height significantly impacts the performance of the test, reinforcing the need for incorporating a new parameter into our analysis, TR_c_, that accounts for the thickness of the membrane, unlike the traditional Damköhler number (Da) which only considers reaction rates and mass transport.

To assess fidelity of the model, two key parameters of the simulation result and experimental data were compared: (i) fluid front arrival time at the test line, and (ii) normalized fluorescence signal at 100 s for membranes of varying thickness. For example, in the 100 μm height configuration at 0 rpm, simulation predicted the fluid front reached the test line at t ≈ 40 ± 2 s, closely matching the experimentally observed time-to-line of 41 ± 3 s (n = 3). Similarly, simulated fluorescence signal formation at 100 s (normalized to a maximum of 1) was 0.85 for biotin–streptavidin and 0.35 for CD79b, compared to experimental fluorescence intensities of 0.82 ± 0.04 and 0.33 ± 0.06, respectively. These comparisons confirm the validity of the COMSOL model under varied channel heights and spin rates.

## 4. Mass Transport-Reaction Kinetics Analysis

### TRc Model Assumption and Limitations

The development of the TRc model in this work involves several simplifying assumptions that should be clarified. First, we assume a steady, laminar flow with constant properties—the volumetric flow rate is taken as constant in time (after initial wetting) and the fluid density and viscosity are constant (appropriate for an aqueous sample). Second, a uniform diffusion coefficient (D) is assumed for the analyte within the membrane; any concentration-dependence or hindrance effects beyond the chosen effective D are neglected. Third, the reaction is modeled as a first-order surface reaction under quasi steady-state conditions, meaning we assume that once the flowing analyte reaches the test line, it instantaneously reacts with available binding sites (up to the limits set by reaction kinetics k) and that the concentrations along the membrane reach a steady profile during flow. In practice, this implies that all analyte that diffuses into the immediate vicinity of the test line has an opportunity to bind (consistent with assuming an excess of binding sites or complete reaction at the surface for simplicity). These assumptions allow us to derive analytical expressions for Da and TRc, but they also impose limitations. For instance, the model does not explicitly account for analyte depletion or product accumulation upstream of the test line, nor for any time-varying effects as the strip initially wicks the sample (our analysis considers the period after flow is established). We also do not include potential non-specific binding or lateral diffusion along the length of the membrane, treating convective flow as the dominant transport mechanism. By adjusting parameters such as flow rate, channel height, reaction rate constant, and diffusivity, the model can be tailored to other LFA systems or surface-based assays beyond the specific platform studied here. In essence, TRc provides a dimensionless measure of the reaction–transport interplay that can be used to compare different assay designs or operational conditions, even though absolute values may vary with system-specific factors. Future work can relax some of these assumptions (for example, incorporating transient wicking or partial analyte depletion) to further refine the model, but within the scope of our analysis the above assumptions hold and define the conditions under which TRc effectively characterizes the system.

The two major parameters which affect the flow rate in the NC membrane are membrane’s porosity and pore size. The pore size is typically classified either via the bubble point of the NC membrane or via pore size distribution.

The bubble point measurement methodology was developed by Millipore Sigma [42]. The bubble point is the minimum pressure required to pass air through a wetted NC membrane. Thus, a higher bubble point refers to lower pore size and hence is inversely proportional to the pore size rating of the membrane.

Pore size distribution is another important parameter that determines which size of pores are most numerous in the NC membrane and whether the smaller pores are more in number or larger pores.

The average pore size *r* of a membrane affects the capillary flow rate via created capillary pressure *P_c_* and hydrodynamic resistance *R_hyd_* presented by the Young–Laplace equation [48,52,53,54,55] below:

(8) $$R_{hyd}=\frac{32.43\mu L}{r^{4}},$$

(9) $$P_{c}=\gamma cos\theta \frac{1}{r},$$

where *L* is the membrane’s length, μ is the viscosity of liquid, γ is coefficient of surface tension of the liquid, and θ is the contact angle.

The porosity of the NC membrane is presented as the percent (%) of the volume of the air pores with respect to the total volume of the membrane.

While the lateral flow through the membrane is represented by Equations (8) and (9) above, the transverse mass transport in the membrane (i.e., through the thickness of the membrane) is carried out primarily by diffusion characterized by an effective diffusion coefficient [53]:

(10) $$D=\theta_{g}\Gamma D_{m}.$$

where $D_{m}$ is the free space coefficient for diffusion, $\theta_{g}$ is the fraction of bulk porous medium that is occupied by gas, i.e., volumetric gas content (dimensionless), and Γ is a tortuosity (dimensionless). In the simulation, the fluid diffusion coefficient (D1 in Table 1) is set to 3 × 10^−11^ m^2^/s. This value is typical for diffusion in porous media (and significantly smaller than the diffusion coefficient in bulk water due to the membrane’s structural constraints)—see Section S6.

The Damköhler number, $D_{a}$ [54,55], for reaction (11) is described by Equation (12) below:

(11) $$EuStr+BSAb(ads)\overset{Ka/Kd}{↔}Cpx(ads).$$

(12) $$D_{a}=\frac{Rateofreaction}{Rateofconvectiontransport}=\frac{-r_{Ao}V}{F_{Ao}}=\frac{kC_{Ao}C_{Bo}V}{C_{Ao}\nu_{o}},$$

(13) $$D_{a}=\frac{kC_{Bo}V}{\nu_{o}}=kC_{Bo},$$

where EuStr concentration is $C_{Ao}$, BSAb(ads) concentration is $C_{Bo}$, $r_{Ao}$ is the reaction rate of the process described by Equation (4) in m/s, V is the volume of the channel above the reaction surface in m^3^, $F_{Ao}$ is the molar flow rate at which reactant A is introduced into the system (in M), k is the reaction rate constant (1/(M·s)), $\nu_{o}$ is the volumetric flow rate of the liquid sample entering the microchannel in m^3^/s, and t_res_ is the mean residence time in s (see Equation (23) below).

In Equation (12), the denominator was used as convection/advection only ($D_{a}$ only instead of Peclet number), since, for particles of 200 nm size, advection dominates diffusion [56]. The conversion factor X in Equation (14) defines the ratio of the amount by analyte concentration after it has been consumed at the test line to produce the signal (we assume for simplicity that all the species at the test line have been reacted with the analyte) with respect to the original analyte concentration:

(14) $$X=\frac{C_{Ao}-C_{Bo}}{C_{Ao}}.$$

From the reaction system, V was defined as the volume flowthrough:

(15) $$V=\frac{XF_{Ao}}{{kC_{Bo}C}_{Ao}}.$$

The volumetric flow rate, *v*_o_, represents the flow of the solution entering the volume (microchannel containing the reacting surface of the test line) and is defined as the product of the flow channel’s cross-sectional area and the liquid’s average velocity. In this system, *v*_o_ can be determined experimentally or approximated using system-specific flow rates provided in prior research [54,55]. We assumed a steady flow profile with constant density. Additionally, for a constant density flow,

(16) $$\frac{V}{\nu_{o}}=\frac{C_{Ao}-C_{Af}}{kC_{Af}C_{Bf}},$$

where $C_{Af}\wedge C_{Bf}$ are final concentrations of EuStr flowing past the test line and of the test line species BSAb(ads), respectively. These concentrations are derived by solving the system’s governing reaction-diffusion equations under steady-state conditions. The system assumes a first-order reaction kinetics model where the concentrations at the end of the line are functions of the initial concentration, flow rate (*v_o_*), and reaction rate constant (*k*). Substituting V and X from Equations (13) and (14) into Equation (16), we obtain

(17) $$\frac{X}{k\left(1-X\right)\cdot (C_{Ao}-X\cdot C_{Bo})}.$$

Solving for X and putting $D_{a}$ using (12), and defining x as $C_{Ao}/C_{Bo}$:

(18) $$X=\frac{1}{2}+\frac{1}{2x}+\frac{1}{2D_{a}x}-\sqrt{{\left(\frac{1}{2}+\frac{1}{2x}+\frac{1}{2D_{a}x}\right)}^{2}-\frac{1}{x}}$$

Equation (18) was used to calculate the $D_{a}$ values provided for different spin. Higher conversion signals result in higher $D_{a}$ values where reaction kinetics dominates mass transport.

Modifying $D_{a}$ to include the role of channel height for the surface reaction (rather than bulk reaction) leads us to introduce a new Transport Reaction constant ${TR}_{c}$ that is applicable for lateral flow assays (and other types of surface reaction affinity assays) and not just for bulk reactions. In Equation (19), the Transport Reaction constant also represents the rate of reaction divided by the rate of convection transport (as in Equation (12)), but the reaction volume V is not all the volume of the channel, but only the volume of the boundary layer adjacent to the test line. If the test line has length L (along the length of the membrane) and width w and the boundary layer has thickness $\delta_{m}$, then the volume of the reacting sample V = $\delta_{m}\cdot L\cdot w$.

To adapt Da for surface-bound assays with finite film thickness, we define the reacting control volume as the diffusion-reachable layer above the line, V = $\delta_{m}\cdot L\cdot w$, rather than the entire film h·L·w. Thus, the transport–reaction constant is

(19) $${TR}_{c}=\frac{reaction\;rate}{convection\;transport\;rate}=\frac{k\;c_{bulk}\;\delta m\cdot L\cdot w}{\;\dot{V}}=\frac{-r_{Ao}V}{F_{Ao}}\;=\frac{kC_{Ao}C_{Bo}\delta_{m}\cdot L\cdot w}{C_{Ao}\cdot \nu_{o}}=\frac{kC_{Bo}\delta_{m}\cdot L\cdot w}{C_{Ao}\cdot \nu_{o}}.$$

Here, k is the apparent surface rate constant (1/s), $c_{bulk}$ is the average analyte concentration above the test line, $\dot{V}$ is the volumetric flow through the control section, and $\delta_{m}$ is the effective boundary-layer thickness accessible within the analyte residence time.

If the channel height is h, then the volume V used in the conventional Damköhler number (Da) is h·L·w; therefore, there is a direct correspondence between ${TR}_{c}$ and $D_{a}$:

(20) $${TR}_{c}=Da\frac{\delta_{m}}{h}.$$

**Figure 7:**
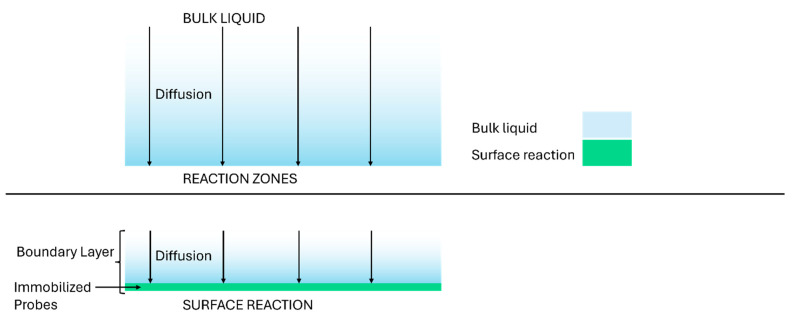
Comparison of bulk reactions (top) and surface reactions (bottom), illustrating the role of diffusion boundary layers and channel geometry.

For shallow channels (${h\approx \delta }_{m}$), ${TR}_{c}\approx D_{a}$, aligning with the traditional definition of the Damköhler number. Conversely, in deep channels (${h\gg \delta }_{m}$, most of the analyte passes through the test chamber unreacted; consequently, TRc will be significantly smaller than $D_{a}$. This is shown schematically in Figure 7.

Utilizing TRc instead of Da for surface reactions will prevent overestimation of the system performance in cases of deep channels while remaining consistent with the traditional Da formulation for the system with shallow reacting volume (such as for lateral flow strips with negligeable thickness of liquid layer above the lateral strip).

To further refine the relationship between the Damköhler number ($D_{a}$) and boundary layer-dominated transport processes, we incorporate a more precise definition of the boundary layer (δ), which depends on the dwell time of the analyte above the reacting strip rather than the classical hydrodynamic definition where concentration gradients determine the boundary layer thickness. This refined perspective recognizes that analyte molecules take a finite time to diffuse from the bulk fluid to the reacting surface, which can be estimated using the Einstein–Smoluchowski equation:

(21) $$D=\frac{\delta^{2}}{2t},$$

where δ represents the distance between the analyte molecule and the reacting surface, *D* is the self-diffusion constant (since we assume that the analyte is not significantly depleted by the surface reaction), and t is the time for the analyte to reach the reacting surface. Given that the moving liquid front advances with velocity υ over a reacting strip of length L, the analyte’s effective residence time above the reacting surface is

(22) $$t=\frac{L}{\upsilon }$$

We estimate δ from the diffusion distance achievable during the analyte’s dwell time above the line. With line length L and average front velocity υ, the residence time is $t_{res}=\frac{L}{\upsilon }$. Substituting this into the diffusion equation, the diffusion boundary layer thickness is expressed as

(23) $$\delta \;\approx \;\sqrt{2Dt_{res}}=\sqrt{\frac{2DL}{\upsilon }}.$$

**Figure 8:**
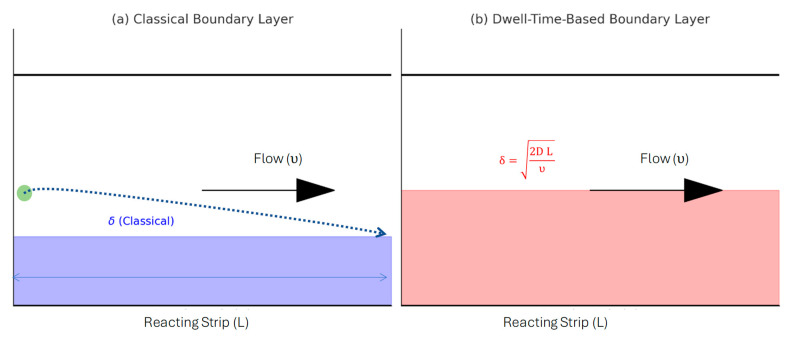
Comparison of Classical and Dwell-Time-Based Boundary Layer Definitions. (**a**) The classical concentration boundary layer is defined as an area where significant concentration gradient exists. (**b**) The dwell-time-based boundary layer is redefined based on the time available for analyte to reach the reacting surface. Arrow shows the flow direction of the fluid meniscus.

Using Einstein–Smoluchowski, this refined boundary layer definition allows us to update the transport-reaction constant (${TR}_{c}$) formulation to explicitly account for diffusion constraints:

(24) $${TR}_{c}=\frac{\delta }{h}\cdot D_{a}=\left(\sqrt{\frac{2DL}{\upsilon }}/h\right)\cdot D_{a},$$

where h is the channel height, D is the analyte’s diffusion constant, L is the strip length aligned with the flow, and υ is the fluid velocity. This modification clarifies that in channels where h≫δ, only a small portion of the analyte participates in the reaction, while for shallow channels where h≈δ, bulk and surface reactions become indistinguishable, leading to $D_{a}\approx {TR}_{c}$. The difference between concentration-based thickness of the boundary layer and the dwell-time based boundary layer thickness (adopted by the present study) is presented in Figure 8.

Table 2 presents comparison of the transport–reaction constant (TRc) to other reaction–transport merit numbers. Unlike the Damköhler number (Da), which treats the entire liquid film as reactive volume, TRc explicitly focuses on the active surface of the considered volume, preventing sensitivity overestimation for deep channels. The Péclet number (Pe) indicates advective vs. diffusive dominance but does not directly address surface capture efficiency. The Sherwood number (Sh) relates surface flux to bulk concentration but requires estimating a mass-transfer coefficient k_m_, which is ill-defined for advancing porous fronts. TRc, instead, uses directly measurable or available values (strip length (L), front velocity (u), channel height (h), diffusion coefficient (D)) and can be used to categorize assay operation into various regimes (TRc > 1: reaction-limited; 0.1 < TRc < 1: mixed regime; TRc < 0.1: transport-limited).

The introduction of TRc provides a practical tool for designing and optimizing flow-modulated surface-based assays. It enables direct comparison across geometries, flow conditions, and reaction kinetics, and can guide assay optimization for enhanced signal output, especially in platforms where film height and dwell time are adjustable. TRc can be applied beyond this LFA-CD system to any surface-based biosensor with geometry-constrained flow, such as microchannels, dipsticks, or 3D-printed diagnostic chips.

As an example of useful insight TRc would provide compared to Da, consider an LFA-CD system with a 3 mm wide strip and a membrane film thickness of 100 µm versus similar membrane with thickness of 300 µm. While the taller microchannel (equivalent to the thicker liquid film) allows more sample volume to be processed, it simultaneously reduces the TRc value, indicating lower analyte residence time near the reactive surface. In contrast, reducing the film height increases TRc, improving signal generation for slower-binding interactions like CD79b. Thus, we believe that TRc is superior to Da as a merit number in optimizing efficiency of surface based microfluidic assays.

## 5. Results and Discussion

### 5.1. Sample Volume Influence on the Detection Signal Strength

**Figure 9:**
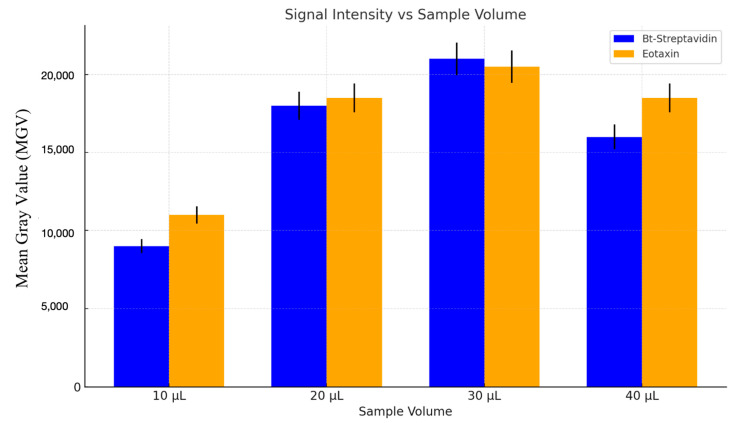
Comparison of the COMSOL simulation results and optical signal observed at the test line for various lateral flow assay sample volumes on a centrifugal microfluidic (CD) platform rotating at 500 revolutions per minute.

Figure 9 presents a comparison of experimental and COMSOL simulation results for the grayscale signal intensity at the test line in a lateral flow assay, conducted at a spin rate of 500 rpm. The test was performed with different fluid volumes by incrementally adding 10 μL steps to the inlet chamber, ranging from 10 μL to 40 μL. The signal at the test line was plotted against the sample volume.

Both experimental and simulation results show that the signal intensity increases with increasing sample volume, reaching a peak at 30 μL, and then decreasing at 40 μL. The COMSOL simulation follows the same trend but with slightly higher predicted values. The decrease in signal intensity at 40 μL is consistent with the observation of other researchers such Xia et al. [40] and attributable to higher flow rate due to stronger capillary pull of the larger sample volume. Mechanistically, larger initial volumes increase capillary head, transiently elevating u and reducing t_res_ as the front crosses the test line, thereby lowering δ and TRc. We observe optimal signal near 30 µL where increased analyte delivery is not yet offset by residence-time loss.

### 5.2. CD Spin Speed Influence on the Detection Signal Strength

It is expected that when the liquid sample dwells on the test line, a higher fraction of analytes will bind to the immobilized probes on the reacting surface compared to an assay where the sample flows past the test line rapidly. Faster spinning of the disc slows down the radial advancement of the capillary meniscus, as demonstrated in previous experiments [32]. Therefore, increasing the CD spin rate is expected to enhance the signal intensity at the test line by extending the analyte residence time. This hypothesis is validated experimentally.

**Figure 10:**
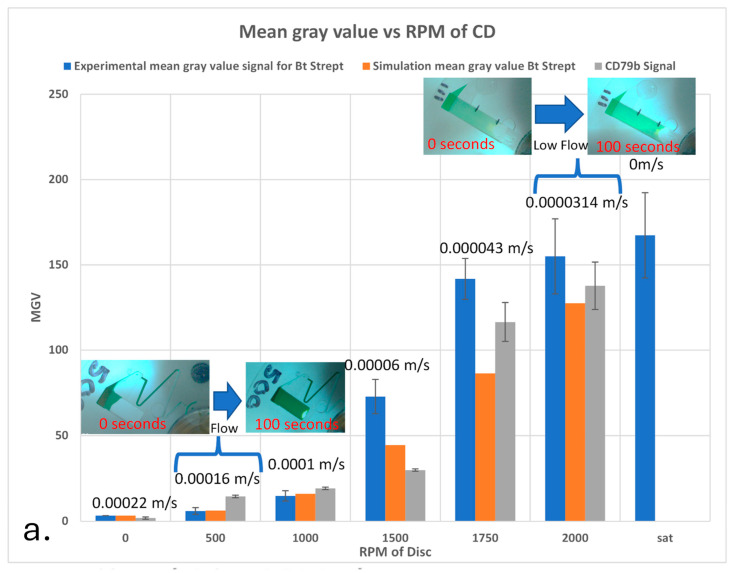

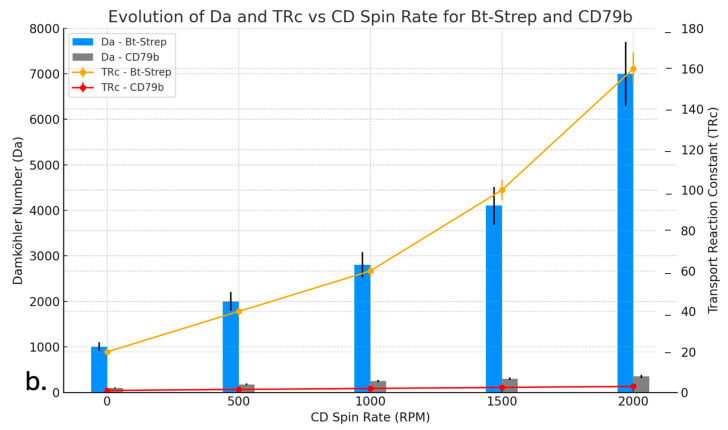
(**a**) Comparison of experimental and COMSOL simulation results for Mean Gray Value (MGV) at different CD spin rates (rpm). The blue bars represent experimental MGV for the biotin–streptavidin (Bt–Strept) interaction, while the orange bars correspond to the simulated MGV. Inset images illustrate analyte flow at several disc spin rates. The saturation (Sat) condition represents the maximum possible signal obtained when the NC membrane is fully immersed in analyte for 4 min. (**b**) Damköhler number (Da) variation with CD spin rate for fast- and slow-reacting systems. The blue bars represent Da values for fast-reacting biotin–streptavidin (Bt–Strept), while the orange line represents Da values for the slower-reacting CD79 antigen–antibody system. The Da numbers for Bt–Strept are indicated on the left vertical axis; for CD79b, they are on the right vertical axis to facilitate legibility.

Figure 10 presents both experimental and simulation data illustrating the effect of CD spin rate on the signal intensity at the test line in a lateral flow assay. In Figure 10a, the experimental results (blue bars) display the variation in Mean Gray Value (MGV) at the test line for different CD spin rates (given in rpm). The signal intensity was simulated by mapping analyte concentration to experimental calibration curves, which establishes a relationship between analyte concentration and grayscale intensity. The experimental flow velocity at the test line match the trends presented by simulations performed by Shen et al. [36]. The deviation from the simulation at higher rpm is likely due to slight ballooning of the disc where fluid inside the channels is under increased centrifugal force at higher rpm. This ballooning slows down the rate of advancement and leads to higher observed signal intensity when compared to simulations.

Inset images in Figure 10a illustrate the fluid movement within the CD membrane at different time points. At 2000 rpm, the advancement of the liquid meniscus in NC membrane stops, allowing the analyte to dwell infinitely at the test line, transitioning the kinetics from a mass transport-limited to a reaction-limited regime.

The saturation (Sat) condition in Figure 10a represents an intensity level where the NC membrane was fully immersed in analyte and stirred for 4 min, yielding the maximum possible signal.

Figure 10b presents the variation in the Damköhler number (Da) as a function of CD spin rate. The blue bars represent Da values for fast-reacting biotin–streptavidin pair (Bt–Strept), while the orange line represents Da values for the slower-reacting CD79 antigen–antibody system. Since CD79 reacts much slower, mass transport should have a greater influence on its signal intensity. Slower reactions mean that dwell time at the test line plays a larger role in enhancing the signal.

At ≥1500 rpm the measured MGV exceeds simulation. We attribute this to slight cover-film deflection (±25–60 µm by dial-indicator at 1800 rpm), which increases hydraulic resistance, slows the front locally, and effectively raises t_res_. The discussion of cover-film deflection of the disc under the increased spin rate is provided in Section S7.

### 5.3. Speed of Reaction Kinetics of Various Analytes and the Height of Microfluidic Channel Influence on the Detection Signal Strength

To experimentally validate the relationship between microchannel height and reaction efficiency, lateral flow assays were performed using a centrifugal microfluidic (CD) platform. Microchannels with controlled heights of 100 μm, 200 μm, and 300 μm were used, achieved by milling polymer grooves to specific depths for positioning nitrocellulose (NC) membrane strips [57].

**Figure 11:**
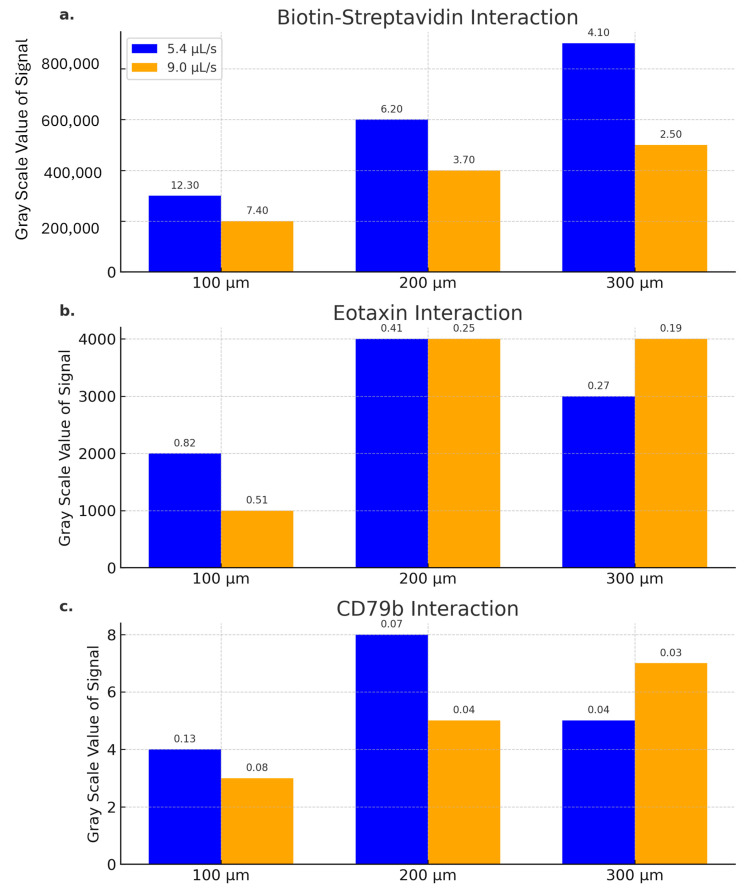
(**a**–**c**) bar plots showing the signal variation for different flow rates (5.4 μL/s and 9.0 μL/s) and several microfluidic channel heights (100 μm, 200 μm, 300 μm) for biotin–streptavidin, Eotaxin, and CD79b interactions, respectively. The Transport Reaction Constant (TRc) values are displayed at the top of each bar to indicate the relationship between reaction rates, film thickness, and mass transport effects.

The bar plots in Figure 11a–c depict the gray-scale signal intensities for three analytes—biotin–streptavidin, Eotaxin, and CD79b—across different channel heights at two flow rates (5.4 μL/s and 9.0 μL/s). Comparing the observed signal strength corresponding to various thicknesses of liquid film above the NC membrane, speed of advancement of the liquid front along the NC membrane, and the type of analyte several important trends can be noted. For fast-reacting reagents such as biotin (in biotin–streptavidin pair), the increase in thickness of liquid film above the NC membrane allows larger amount of analyte to react and thus the signal strength will grow together with the height of the liquid film. For the same biotin–streptavidin pair, higher disc rate and slower meniscus flow increases the dwell time above the reacting surface and results in a stronger observed signal. For slower-reacting species such as Eotaxin and CD79b, we observe the drop in signal as the thickness of the liquid film increases from 200 micron to 300 micron. It likely signifies that centrifugal force pushes some analytes down the liquid film (the centrifugal force is higher for 5.4 μL/s than for 9.0 μL/s since the centrifugal force slows down the capillary force-based advancement of liquid meniscus) before the analytes have a chance to react with the test surface.

**Figure 12:**
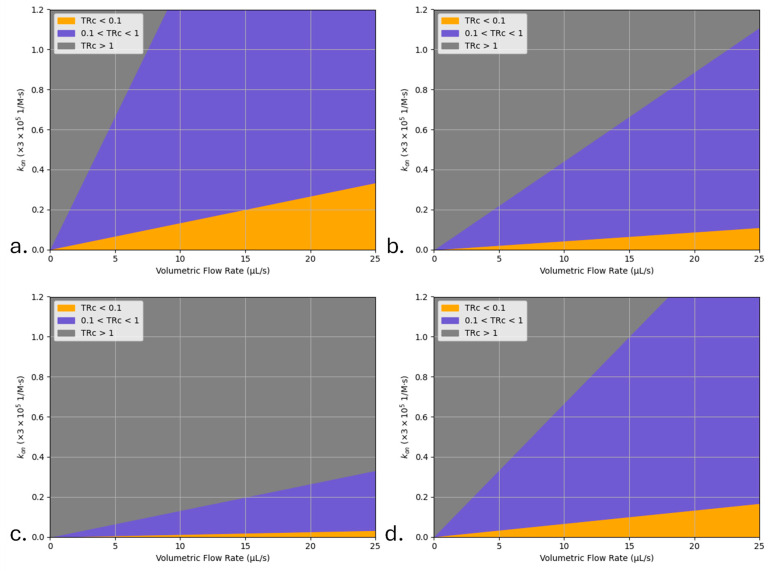
The effect of channel height, reaction kinetics, and width of the test line on the resulting TRc values. The diffusion coefficient (D) of 6.2 × 10^−11^ m^2^/s and the bulk concentration of 5 × 10^−6^ M are assumed. (**a**) TRc distribution for a channel height of 100 µm and test line width of 1 mm; (**b**) TRc distribution for a channel height of 300 µm and test line width of 1 mm; (**c**) TRc distribution for a channel height of 1 mm and test line width of 1 mm; and (**d**) TRc distribution for a channel height of 100 µm and test line width of 2 mm. The overlapping color regions in (**a**–**d**) do not affect scientific interpretation, as the TRc regime boundaries remain distinct. To improve visual clarity for readers, color contrast and legend placement were adjusted, ensuring that each TRc zone (transport-limited, mixed, and reaction-limited) remains visually and conceptually separated.

Figure 12 illustrates the Transport Reaction Constant (TRc) dependence on flow rates and channel heights of the system. In order to obtain the values for TRc, Da is calculated first, using Equation (13) and then TRc is calculated, using Equation (24). For purposes of these calculations, the diffusion coefficient (D) was taken to be 6.2 × 10^−11^ m^2^/s and the bulk concentration was taken to be 5 × 10^−6^ M. While specific values will be different for different systems, it will be instructive to review the effects of the channels height, reaction kinetics, and the flow rate on the overall TRc values.

Three distinct TRc regimes are identified:

1. TRc > 1: Reaction rate dominates over mass transport.
2. 0.1 < TRc < 1: Reaction and transport effects are balanced.
3. TRc < 0.1: Mass transport limits reactant access to the active surface [58].

The plots in Figure 12a–c illustrate how an increase in channel height (thickness of liquid film) shifts TRc toward lower values, particularly at high flow rates, while the plot in Figure 12d further demonstrates that the increase in test line width increases the available reaction surface, thereby shifting the system away from mass transport limitations.

These findings emphasize the critical role of microfluidic geometry in optimizing lateral flow assay performance, particularly for analytes with slower diffusion rates. Balancing flow rate, film thickness, and reaction kinetics is essential for maximizing signal strength and minimizing mass transport limitations in biosensor applications and for chemical and biological assays.

The channel heights of 100 μm, 200 μm, and 300 μm were deliberately selected to represent a range from approximately the thickness of a standard LFA membrane up to about three times that thickness. In typical lateral flow strips, the nitrocellulose membrane (often backed with a plastic support) has an overall thickness on the order of a few hundred micrometers. For example, a common backed LFA membrane is around 240–270 μm thick. Thus, a 100 μm channel height in our experiments approximates a scenario with essentially no additional fluid layer beyond the membrane itself, whereas 300 μm provides a substantially thicker fluid layer above the membrane (mimicking a deep microfluidic chamber). Channel heights in the 100–300 μm range are also characteristic of many microfluidic devices, making our choices representative of standard lab-on-chip geometries. By examining 100, 200, and 300 μm heights, we capture the transition from a regime in which nearly all analyte is immediately adjacent to the reactive surface (the 100 μm case) to one in which a significant portion of the analyte resides farther from the surface (300 μm case). These three heights therefore enable us to systematically investigate how increasing the vertical dimension of the flow path influences mass transport and reaction kinetics in the LFA, and to identify an optimal channel geometry for assay performance.

## 6. Conclusions

The presented work demonstrated that the combination of lateral flow assays with centrifugal microfluidics allows enhancement of assays’ sensitivity (demonstrated via the increase in the strength of the fluorescent signal) by increasing the dwell time of the analyte at the test line as well as by the ability of passing larger volume of analyte through lateral flow assay strip. The rate of the advancement of liquid front in the radially positioned nitrocellulose strip is retarded by the centrifugal force generated on spinning disc and thus the dwell time of the liquid front above the test line is increased. Furthermore, placing the lateral flow strip on the rotating disc enables the solution reaching the end of the strip to drain into a waste reservoir. Once the end of the test strip is drained, the capillary force draws additional sample through the NC, thereby increasing the probed volume of the sample.

The finite element model of the lateral flow assay placed on the rotating platform was performed on the multiphysics simulation platform COMSOL. The simulation reflected the effect of the change in three parameters: the sample volume, the angular velocity of the spinning disc, and the height of the microchannel. To validate the simulation, a set of experiments was performed using a nitrocellulose membrane radially integrated into a centrifugal microfluidic platform. The following parameters were varied: sample volume, from 10 μL to 40 μL in 10 μL increments; angular velocity, from 0 to 2000 rpm; reaction kinetics, by comparing fast-reacting reagents such as biotin–streptavidin with slower-reacting CD79b protein–antibody pairs; and microchannel height, tested at 100, 200, and 300 microns.

A Transport Reaction constant (TRc), a novel dimensionless parameter that refines the characterization of reaction–transport dynamics in surface-based assays is introduced. Unlike the traditional Damköhler number (Da), which was designed to be used for bulk reactions, TRc accounts for channel dimensions and test line width, making it particularly relevant for surface-based reactions in microfluidic systems. The study identifies three critical flow regimes based on TRc values, providing a framework for optimizing reaction and transport kinetics in Lateral Flow Assays.

## Figures and Tables

**Table 1:** COMSOL simulation variables to simulate a rapid detection test modified from a model simulation from COMSOL [50].

Name	Value	Description
L0	39.6 mm	Paper strip height
W0	2.96 mm	Paper strip width
th	0.63 mm	Paper strip thickness
gamma	0.0723 N/m	Surface tension
theta	50 deg	Contact angle
Rc	13.5 × 10^−6^ m	Pore radius/HF090 membrane
pec	44,492 N/m^2^	Entry capillary pressure
lp	2	Pore size distribution index
por	0.8	Porosity
K	3.58 × 10^−12^ m^2^	Permeability
rho_air	1 kg/m^3^	Air density
rho_water	1000 kg/m^3^	Water density
mu_air	1.76 × 10^−5^ Pa·s	Air viscosity
mu_water	0.001 Pa·s	Water viscosity
omega	100 rad/s	Angular acceleration
Rout	5 cm	Strip outer-end location on disc
Rin	Rout-L0	Strip inner-end location on disc
k1	13.5 × 10^8^ m^3^/s·mol	Kon
c0	2.3 × 10^−4^ mol/m^3^	Inlet antibody concentration
c0ads	5 × 10^−7^ mol/m^2^	Adsorbed species surface concentration, conjugate pad
D1	3 × 10^−11^ m^2^/s	viscosity
k2	k1/10	Koff
N_a	6.022 × 10^23^ mol^−1^	Avogadro’s number
d_pa	1.3 × 10^−5^ m	Particle diameter

**Table 2:** Comparison of dimensionless metrics for reaction–transport microfluidic systems. ✓/✗ means yes or no.

	Damköhler (Da)	Péclet (Pe)	Sherwood (Sh)	Transport–Reaction (TRc)
What does each merit number represent?	Reaction rate vs. advective transport	Convective vs. diffusive transport	Mass transfer coefficient vs. diffusion	Effective surface reaction efficiency under constrained film thickness
Does it explicitly include the liquid film height?	✗	✗	✗	✓
Surface vs. bulk assay applicability	✗ Bulk reactions with limited applicability for surface reactions	✗ Bulk transport	✗ Mass flux at interface, no reaction kinetics	✓ Applicable for surface-based reactions
Does it include the dwell time over active surface?	✓ Indirectly via flow rate	✗	✓ Indirectly, via convective mass transfer coefficient	✓ (t_res_ = L/u)
Can it be used as a guideline for surface assay optimization?	✗	✗	✗	✓ Consideration of three TRc regimes (>1, 0.1–1, <0.1)

## Data Availability

The original contributions presented in this study are included in the article and its Supplementary Material. Additional raw data, including COMSOL simulation files and fluorescence image datasets, are available from the corresponding author upon reasonable request.

## Supplementary Materials

The following supporting information can be downloaded at: https://www.mdpi.com/article/10.3390/s25206271/s1, including: Figure S1: Alternative membrane designs, including the left elbow and L-shaped configurations. Figure S2: Quality control data for europium (Eu) nanoparticle probes, showing hydrodynamic diameter distributions and ζ-potential shifts post-conjugation. Figure S3: Calibration curves for the detection of Biotin-Streptavidin, Eotaxin, and CD79b on the LFA–CD platform. Figure S4: Plots showing the conversion of spin rate (rpm) to front velocity and the measured cover-film deflection as a function of spin rate. Table S1: Conjugation quality control results, including conjugation yield, DLS diameter, and ζ-potential for various Eu-nanoparticle probes. Table S2: A summary of calibration fit parameters and analytical performance figures of merit, such as LOD and LOQ. Table S3: Detailed list of reagents, materials, and instrumentation suppliers. Table S4: A comparison between the Transport Reaction Constant (TRc) and the Damköhler Number (Da) metrics. Additional Information: The supplement also includes the detailed protocol for the Europium Conjugation Kit, and the methodology for estimating the diffusion coefficient using the Stokes-Einstein equation.
