# Supplementary material for: Interplay of the Mass Transport and Reaction Kinetics for Lateral Flow Immunoassay Integrated on Lab-on-Disc
Source: Sensors (Basel). 2025 Oct 10;25(20):6271. doi: 10.3390/s25206271 (PMC12567277; doi:10.3390/s25206271)
Supplement: Supplementary file 1 [file sensors-25-06271-s001.zip › sensors-3875847-supplementary.pdf]

SUPPLEMENTARY INFORMATION

S1: Alternative membrane designs

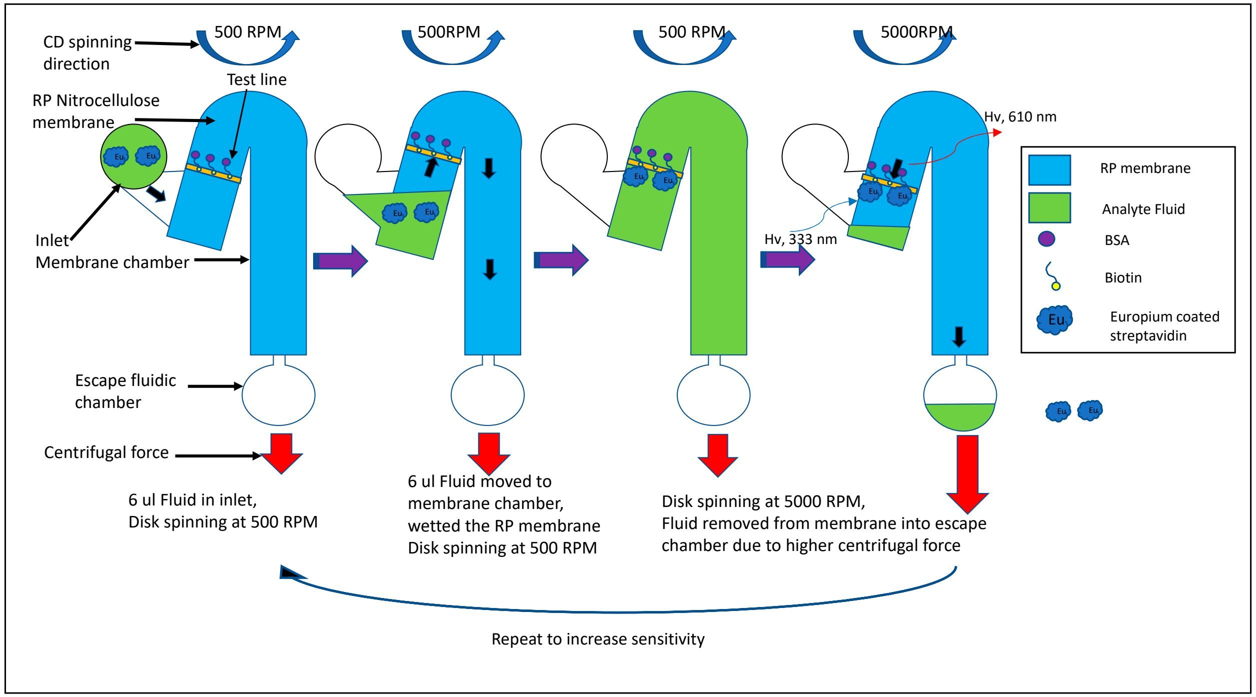

Figure S1 (a). The left elbow membrane design.

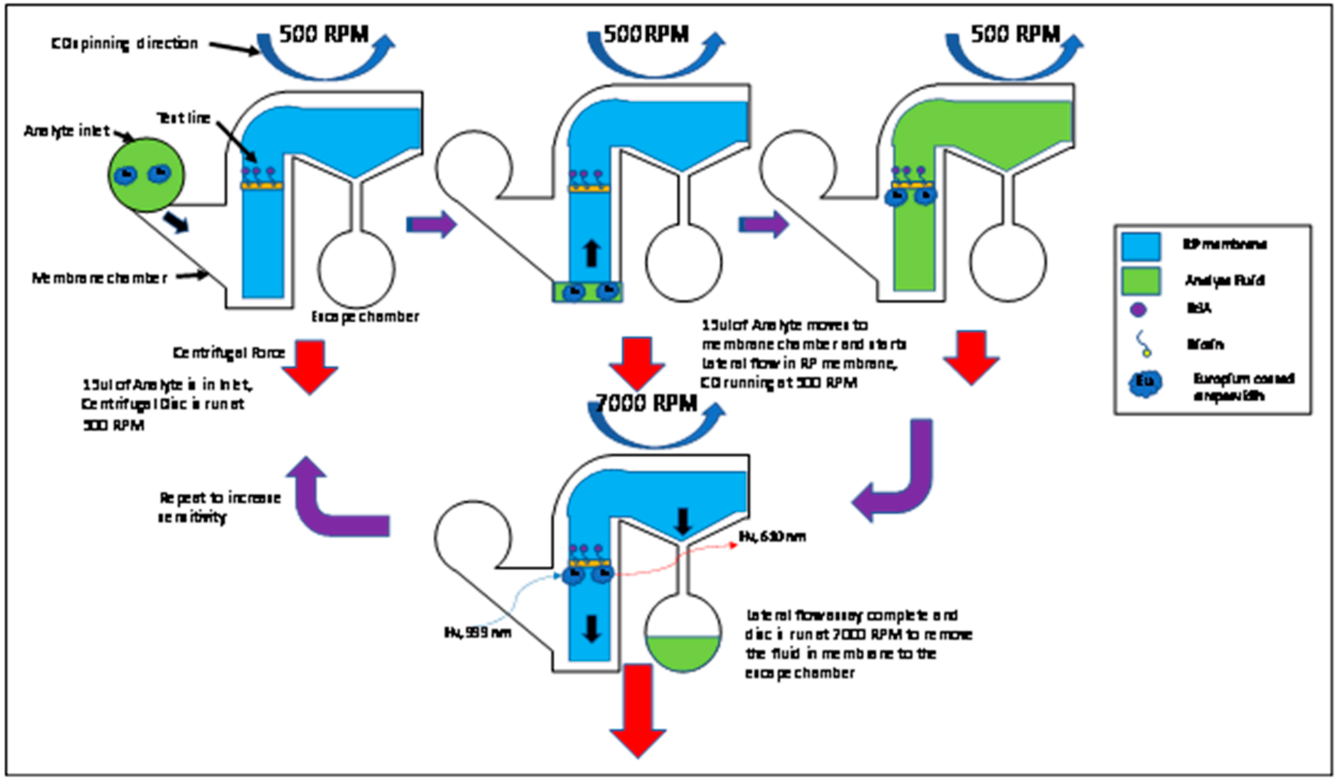

Figure S1 (b). The L-shaped membrane design.

1. Left Elbow Membrane Design (Figure S1a)

In this design, **fluid is introduced into the membrane from the shorter side** at a **lower spin of the centrifugal disc**. The fluid then **permeates the membrane**, saturating all the **pores in both the longer and shorter stems** due to the **lower centrifugal force**.

- Once the initial permeation occurs, the **CD is spun at a higher speed**, **significantly increasing the centrifugal force**.
- This **draws the fluid downward**, and since more pores are in the **longer stem**, it **drains the fluid into the escape chamber**.
- This process can be **repeated** to **enhance the inlet volume throughput** of the lateral flow system.

2. Right Elbow Triangular Design (Figure S1b)

Here, the **fluid enters the membrane through the longer stem**.

- The **CD is initially spun at a lower speed of 500 rpm (revolutions per minute)**, allowing the fluid to **flow and saturate both the longer stem and the wider triangular region** with fluid.
- The **CD is then spun at a high speed of 7000 rpm**.
- Due to more **pores in the wider triangular region**, the fluid **becomes trapped** and attempts to **move downward under centrifugal force**.
- Eventually, the **fluid drains out through the triangular-shaped membrane into the escape chamber**.

Another **method to enhance analyte absorbance** in the triangular region is **replacing it with water gel powder or a hydrogel bag**, which **stores and gradually releases** the analyte when the **CD is spun**.

3. Biotinylated BSA and Fluorescence Measurement

- **Test line: Biotinylated BSA (2 mg/mL)**
- **Analyte: Dilutions of europium-coated streptavidin (Thermo Fisher Scientific)**
- **Detection method: Time-resolved fluorescence**, with
    - **Absorption spectrum at 365 nm;**
    - **Emission at 610 nm;**
    - **Time lag of ~600 μs.**

4. Space Optimization for Lateral Flow on CD

To optimize space, a **variation of this design** allows the **longer part of the membrane to enter the backside of the CD** through a **hole in the acrylic CD at the elbow**. This is achieved by either

- **Folding the membrane**, or
- **Cutting it at an angle**.

This **reduces the required space** at the **front of the CD**, allowing **10-15 lateral flow setups** to be placed **on a single CD**.

S2: Europium Conjugation Kit Protocol

1. Overview

The Europium Conjugation Kit (**ab269889**) facilitates the **rapid and simple conjugation of antibodies or proteins** to high-quality **200 nm europium (Eu) chelate microspheres**. Specially treated to **enhance handling** and **permit covalent attachment**, the **freeze-dried Eu microspheres** enable a **quick initiation** of the conjugation reaction by **reconstituting with the antibody**. This process attaches the antibody to the **treated surface via lysine residues**.

The entire conjugation setup takes **only 30 seconds**, with a **hands-on time of approximately 3 minutes**. Within **35 minutes**, the conjugate is ready for use. The **surface treatment imparts resistance to aggregation**, and the **Eu fluorescence signal enhances sensitivity** in **immunoassays** like lateral flow assays. The conjugation procedure is **minimally dependent on the isoelectric point of the antibody**, and the kit provides **two pH options** (**Reaction Buffers A and B**) for conjugation.

2. Materials Supplied and Storage

Store the kit at -20°C upon receipt.

- Buffers and the Quencher can be stored at **+4°C or -20°C**.
- The kit remains **viable for 1 year** from receipt if components are **not reconstituted**.
- **Avoid repeated freeze-thaws** of reagents.

3. Technical Considerations

3.1. Amount of Antibody to be Conjugated

- The **optimal amount of antibody** depends on **particle size** and **application**.
- **Initial testing at 0.1 mg/mL is recommended**, with flexibility to explore **slightly lower or higher concentrations**.
- For concentrations **above 0.1 mg/mL**, use the **Antibody Concentration & Clean Up Kit for Latex and Europium (ab269889)** to **remove interfering buffer components**.

3.2. Buffer Considerations Before Conjugation

- Efficient conjugation relies on **proper buffer composition**.
- Perform conjugation from stock antibodies at **1 mg/mL** in **10 mM-50 mM MES, HEPES, or MOPS at pH 6-7**.
- The **Antibody Concentration and Clean Up kit (ab269965)** can simplify the purification process.
- Refer to the kit protocol for **detailed buffer preparation steps**.

4. Assay Procedure

4.1. Allow Reagents to Warm

Ensure **all reagents reach room temperature** before use.

4.2. Antibody Dilution

a. Dilute stock antibody to **0.1 mg/mL** with **Reaction Buffer A or B**.
b. Use **45 µL** for **one 200 nm Europium vial**.
c. For **initial tests**, conduct reactions with **both buffers** to **find optimal pH**.
d. Explore different antibody dilutions to **examine varying conjugation efficiency**.

4.3. Conjugation Reaction

a. Add **40 µL of 0.1 mg/mL antibody** to the **200 nm Europium vial**.
b. Reconstitute Eu particles by **pipetting** and **incubate for 15 minutes at room temperature**.

4.4. Europium Quencher Preparation

- Dilute **10x Europium Quencher** with **deionized water**.
- For **one vial**, use **1 mL (1.2 mL 1x Europium Quencher per vial)**.

4.5. Stop the Reaction

- After **15 minutes**, add **1 mL of 1X Europium Quencher**, mix, and invert.

4.6. Quenching and Pellet Formation

a. Quench for **5 minutes**, then **transfer to a microcentrifuge tube** and **spin down the sample**.
b. Remove supernatant and **spin again** for better pellet formation.

4.7. Resuspension of Pellet

- **Tap pellet**, add **40 µL Resuspension Buffer**, and **resuspend thoroughly**.

4.8. Final Conjugate

- The final conjugate consists of **40 µL of 1% solution**.

4.9. Dilution for Application

- **Dilute the conjugate** as needed (**recommended: 0.0025%-0.005%**).
- **Optimize the dilution according to your specific application.**

S3: Conjugation quality control of Europium nanoparticle probes

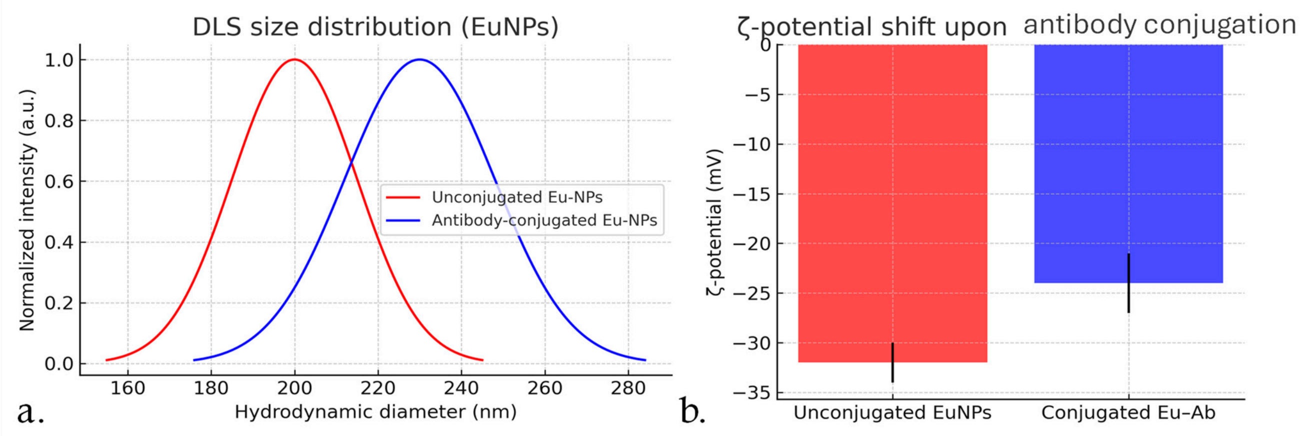

Figure S2. Conjugation quality control of europium (Eu) nanoparticle probes. (a) Dynamic light scattering (DLS) hydrodynamic diameter distributions of unconjugated Eu-NPs (red) and antibody-conjugated Eu-NPs (blue). (b) ζ-potential shift following antibody coupling, confirming successful surface modification.

Table S1. Conjugation QC results.

Probe Type	Input antibody (µg)	Conjugation yield (%)	DLS diameter (nm, mean ± SD)	ζ-potential (mV, mean ± SD)	Working stock (mg/mL)
**Eu–Streptavidin (control)**	—	Manufacturer spec	200 ± 15	–32 ± 2	1.0
**Eu–Anti-Eotaxin**	50	78 ± 5	225 ± 18	–24 ± 3	0.8
**Eu–Anti-CD79b**	50	74 ± 6	238 ± 20	–22 ± 4	0.9
**Eu–Anti-Biotin**	50	80 ± 4	220 ± 17	–25 ± 2	0.85

Notes: DLS shows ~20–30 nm increase post-conjugation, consistent with antibody coating. ζ-potential shift toward neutrality indicates reduced surface charge, confirming conjugation. Yields calculated as (protein recovered after conjugation / input) × 100. Working stocks normalized to ~0.8–1.0 mg/mL.

S4: Calibration curves for analyte detection on the LFA–CD platform

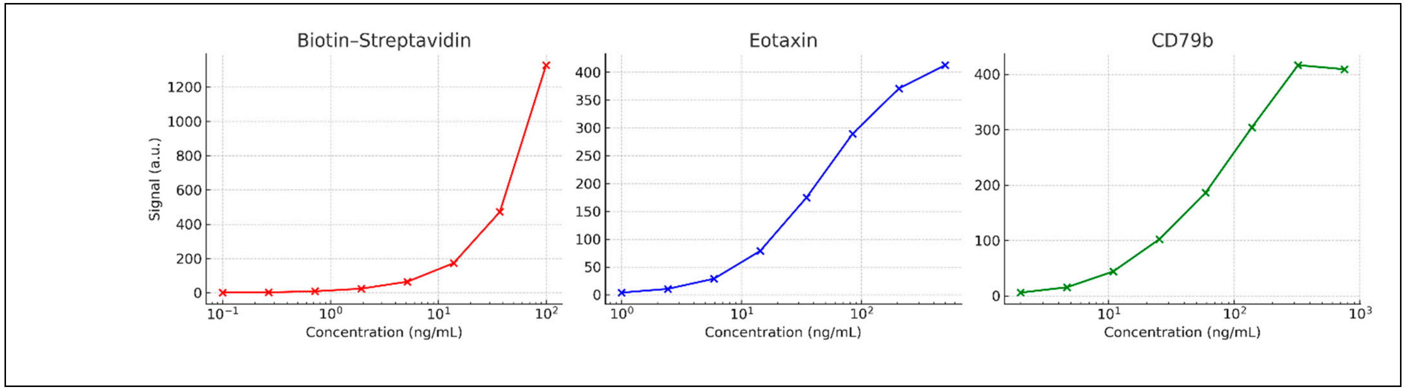

Figure S3. Calibration curves for analyte detection on the LFA–CD platform. Signal (Mean Gray Value, MGV) vs. analyte concentration plotted for (a) biotin–streptavidin, (b) Eotaxin, and (c) CD79b. Each data point is mean ± SD (n = 3). Linear fits shown in low concentration ranges; 4-parameter logistic (4PL) fits shown over full dynamic ranges. The derived analytical figures of merit are summarized in Table S4.

Table S2. Calibration fit parameters and analytical performance.

Analyte	Fit type	Slope (a.u./ng·mL^{-1})	R^2	LOD (ng/mL)	LOQ (ng/mL)	Linear range (ng/mL)
**Biotin–Streptavidin**	Linear	10–15 (typical)	~0.99	**~0.05**	~0.15	0.1 – 100
**Eotaxin**	Linear/4PL	~8–12 (typical)	~0.98	**~0.25**	~0.75	0.5 – 500
**CD79b**	Linear/4PL	~6–10 (typical)	~0.96–0.98	**~0.4**	~1.2	1 – 750

LOD = mean(blank) + 3σ; LOQ = mean(blank) + 10σ. The values representative of Eu-chelate nanoparticle LFAs with test line readout under 365 nm excitation / 610–630 nm emission.

S5: Reagents and Instrumentation Supplier Details

Table S3. Reagents and Instrumentation Supplier Details

**Component/Material**	**Description**	**Catalog Number**	**Supplier**	**Notes**
**Acrylic Disc**	PMMA Clear Scratch- and UV-Resistant Sheet	#8589K43	McMaster-Carr	Base material for CD
**CNC Machine**	Tormach 440 PCNC CNC milling machine	-	Tormach Inc.	Used for micromachining
**NC Membrane**	Hi-Flow Plus HF120	-	Millipore Sigma	Main assay strip material
**Adhesive**	Double-sided adhesive (LFA compatible)	#90445Q	Adhesives Research / Parafix	For membrane bonding
**Sealing Tape**	Single-sided adhesive tape	9795R	3M	For chamber sealing
**Cutter**	Silhouette Cameo 4 Electronic Cutter	-	Silhouette America	To cut NC strips
**Capture Line Printer**	AD3050	-	BioDot Inc.	Prints antigen capture lines
**Roller**	Manual Pressure Roller	B07YDNKSH6	Akiro (Amazon)	Uniform sealing
**Motor**	Brushless DC Servo Motor	SM3450D	Motion USA	Disc rotation
**Motor Controller**	BLDC Servo Controller	EZSV23/EZSV17	AllMotion	Motor control via RS-232
**Sensor**	Optical Sensor (Reflective marker based)	KY-032	Keyestudio / Arduino	Spin-speed monitoring
**Camera**	Tiny Imager / acA800-510uc	-	Basler AG	Captures test line fluorescence

**Strobe Light**	Strobe Sync Light	DT-311A	Shimpo	Image synchronization
**Screen Capture**	Recording Software	-	Bandicam Company	Creates video from camera
**Detection Probe**	Fluoro-Max Europium Streptavidin Particles	29470701010350	Thermo Fisher	Analyte probe
**PBS**	10X PBS, pH 7.4, RNase-free	AM9624	Thermo Fisher	Buffer solution
**Eotaxin Antibody**	Recombinant Anti-Eotaxin	ab133604	Abcam	Capture antibody
**Eotaxin Protein**	Recombinant Human Eotaxin	ab282376	Abcam	Target antigen
**CD79b Antibody**	Recombinant Anti-CD79b	ab134103	Abcam	Capture antibody
**CD79b Protein**	Recombinant Human CD79b	ab153795	Abcam	Target antigen
**Conjugation Kit**	Europium Labeling Kit	-	Abcam	Used for Eu conjugation
**Fluorescence Reader**	Custom Tiny Imager	MU2003/ET615/40m	AmScope	Fluorescence signal capture
**UV Flashlight**	UV Source (365 nm)	-	Temu Inc.	Excitation source

S6: Estimation of Diffusion Coefficient for Simulation

The diffusion coefficient $D = 6.2 \times 10^{-11}\ \mathrm{m}^2/\mathrm{s}$ used in the COMSOL simulation was calculated using the **Stokes–Einstein equation:**

$$D = \frac{k_B \cdot T}{6\pi\eta r}, \quad (26)$$

where

$k_B = 1.38 \times 10^{-23}$ J/K is the Boltzmann constant,

$T = 298$ K is the room temperature,

$\eta = 0.001$ Pa · s is the dynamic viscosity of water,

$r \approx 6.5$ nm $= 6.5 \times 10^{-9}$ m is the **hydrodynamic radius** of the europium nanoparticle, as measured by **dynamic light scattering (DLS)**.

Substituting into the equation

$$D \approx \frac{1.38 \times 10^{-23} \times 298}{6 \times \pi \times 0.001 \times 6.5 \times 10^{-9}} \approx 6.2 \times 10^{-11}\ \mathrm{m^2/s}, \quad (27)$$

the hydrodynamic diameter and ζ-potential of the europium-labeled nanoparticles were verified experimentally using **DLS** and are reported in **Table S1**.

S7: Disc deformation under centrifugal force

For a disc of radius r and angular velocity ω = 2πN/60 (rad·s^{-1}, with N = rpm), the centrifugal pressure difference at radial position r is

$$\Delta P = ½\, \rho\, \omega^2\, (r\_out^2 - r\_in^2), \quad (28)$$

where ϱ is the liquid density, and r_out and r_in are the outer and inner radii of the liquid column. The front velocity u at the test line is measured experimentally from the time-to-arrival of the meniscus:

$$u = \frac{L}{t_{front}}, \quad (29)$$

where L is the distance from inlet to test line and t_front is the measured arrival time at a given rpm.

**Calibration example values (LoD system):**	
**rpm**	**Front velocity u (mm·s^{-1})**

500	0.42
1000	0.84
1500	1.26
2000	1.68

Disc deformation under centrifugal force

Figure S7b contains the plot of tachometer-synchronized stroboscopic image profilometry documenting the deformation of the cover film under specific angular velocity. Above 1500 rpm, the measured mean gray value exceeded simulation predictions. We attribute this to slight cover-film deflection (25–60 µm at 1800 rpm), which increases hydraulic resistance, slows the front locally, and effectively raises the analyte residence time. Profilometry traces confirm a monotonic relationship between rpm and deflection depth.

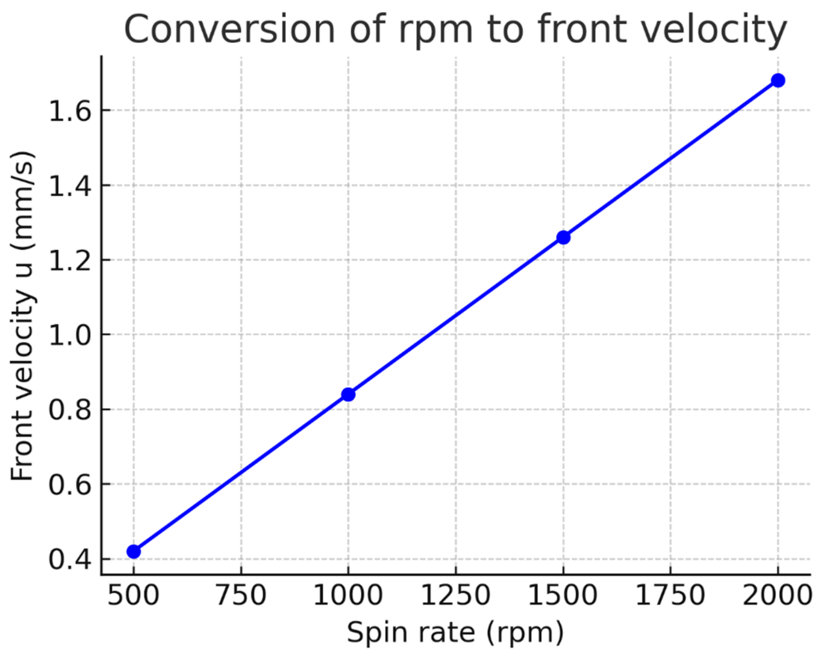

Figure S4a. Conversion of spin rate (rpm) to front velocity (u) in the Lab-on-Disc (LoD) system. Front velocity was derived from time-to-line measurements under stroboscopic observation. The relationship is approximately linear in the operating range (500–2000 rpm).

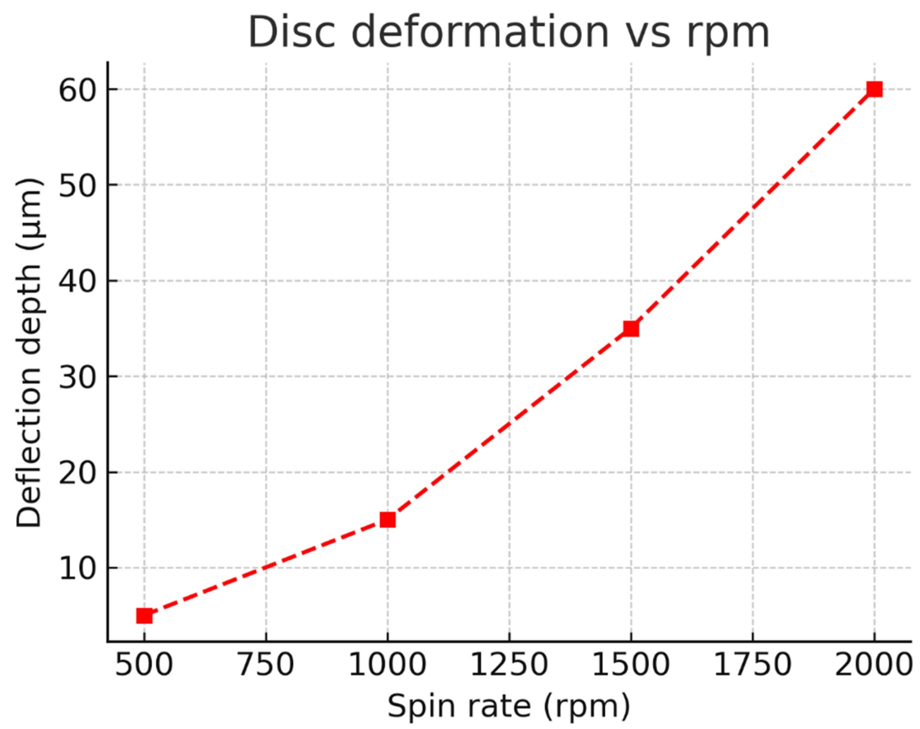

Figure S4b. Profilometry-measured cover-film deflection as a function of spin rate (rpm). Deflection increases monotonically with rpm, reaching 25–60 µm at 1800–2000 rpm. This deformation increases hydraulic resistance and prolongs analyte residence time, consistent with experimental signals exceeding simulation predictions at high rpm.

S8: Comparison of TRc and Da

Table S4: Comparison of Transport Reaction Constant (TRc) vs. Damköhler Number (Da)

**Metric**	**Reflects Reaction Rate Influence**	**Reflects Mass Transport Influence**	**Reflects Liquid Flim Thickness Influence**	**Appropriate for…**
**Da**	☑ Yes	☑ Yes	✕ No	Bulk/ideal reactors
**TRc**	☑ Yes	☑ Yes	☑ Yes	Surface-based microfluidics (LFAs, paper assays)
